# Association of vitamin D receptor genetic variants with therapeutic response in multidrug-resistant pulmonary tuberculosis: a systematic review

**DOI:** 10.3389/fcimb.2026.1692281

**Published:** 2026-02-09

**Authors:** Jaishriram Rathored, Tanushree Budhbaware, Sandesh Shende, Vishwanath Upadhyay

**Affiliations:** 1 Central Research Laboratory and Molecular Diagnostics, Datta Meghe Institute of Higher Education and Research, Sawangi (Meghe), Wardha, Maharashtra, India; 2 Department of Molecular Medicine, Jamia Hamdard (Deemed to be University), New Delhi, India

**Keywords:** genetic variants, multi-drug-resistant tuberculosis (MDR-TB), polymorphism, treatment response, tuberculosis, vitamin D, vitamin D receptors (VDRs)

## Abstract

**Introduction:**

In high-burden nations like India, tuberculosis (TB) continues to be a significant global public health concern. HIV infection, diabetes mellitus, and low socioeconomic status are examples of comorbid illnesses that increase susceptibility to tuberculosis (TB), and the introduction of multidrug-resistant tuberculosis (MDR-TB) has made disease control even more challenging. The time-consuming nature of conventional drug susceptibility testing (DST) emphasizes the critical need for quick biomarkers to forecast treatment outcomes and resistance. Because of their possible impact on host immunity and MDR-TB risk, genetic variations particularly vitamin D receptor (VDR) polymorphisms have drawn attention.

**Methods:**

Studies published between 2000 and 2024 were the subject of an extensive examination of the literature. Relevance led to the selection of 213 articles. Keywords including vitamin D, VDR polymorphisms, MDR-TB, pulmonary tuberculosis, and immune response were used to search databases such as PubMed, Web of Science, and Google Scholar. To guarantee comprehensive coverage, both original research articles and reviews were included.

**Results:**

Low serum vitamin D levels were consistently linked to an elevated risk of MDR-TB and pulmonary tuberculosis (PTB), according to the investigation. Certain VDR polymorphisms have often been associated with altered immunological responses and an increased risk of disease, especially mutant forms like FokI and TaqI. Treatment response and disease progression have also been discovered to be influenced by immunological modulation and dietary variables.

**Discussion:**

These results imply that vitamin D levels and VDR polymorphisms could be useful biomarkers for the diagnosis and prognosis of MDR-TB. Knowing the genetic susceptibility of the host may help develop individualized treatment plans and enhance the management of MDR-TB.

**Systematic Review Registration:**

https://www.crd.york.ac.uk/PROSPERO/view/CRD420261295571, identifier CRD420261295571.

## Introduction

Despite around one-third of the world’s population afflicted and a significant burden concentrated in low- and middle-income nations like India, tuberculosis (TB) continues to be a major worldwide public health concern (Gulumbe et al., 2025). Multidrug-resistant tuberculosis (MDR-TB) has emerged and expanded, making disease control even more difficult. This has resulted in longer treatment times, higher costs, and worse clinical results. Individualized MDR/RR-TB regimens based on patient characteristics, illness severity, previous treatment exposure, and drug-resistance profiles are recommended by current World Health Organization (WHO) guidelines; nonetheless, despite standardized programmatic care, treatment results are still quite diverse (Mase and Chorba, 2019).

TB susceptibility and treatment response are significantly influenced by host-related variables in addition to microbial resistance. Due to its capacity to boost macrophage antimicrobial activity, control cytokine production, and produce antimicrobial peptides like cathelicidin, vitamin D has drawn more attention as an immunomodulatory agent in tuberculosis (Roque-Borda et al., 2025). Low blood vitamin D levels have been linked to an increased risk of pulmonary tuberculosis in a number of observational studies, and vitamin D supplementation as an adjuvant to antitubercular treatment has been investigated in randomized trials with conflicting outcomes (Huang et al., 2016). Eugenol possesses antibacterial and anti-inflammatory properties, however its usage in medication formulations is limited by its cytotoxicity at high doses. Eugenol-based nanoemulsions loaded with several drugs exhibit decreased cytotoxicity, underscoring the need of comprehending how these complexes interact with fundamental human carrier proteins in order to design therapeutic substitutes (Menon et al., 2024).

The vitamin D receptor (VDR), a nuclear hormone receptor encoded on chromosome 12q13–14, mediates the biological effects of vitamin D. The possible impact of common VDR gene polymorphisms, such as FokI, TaqI, BsmI, and ApaI, on TB susceptibility and immunological response has been extensively studied (Usategui-Martín et al., 2022). These single-nucleotide variations may change host immune modulation, downstream gene transcription, and receptor activation. According to population-based research and meta-analyses, ethnicity, environmental exposure, and baseline vitamin D status all have an impact on the diverse relationships between VDR polymorphisms and tuberculosis risk (Tourkochristou et al., 2023).

TB outcomes are further influenced by host genetic variables, malnutrition, poverty, overcrowding, and comorbidities including diabetes and HIV. Patients with active tuberculosis frequently have vitamin D insufficiency, which may be the result of both inadequate diet and metabolic abnormalities brought on by the illness. Due to their cheap cost and biological plausibility, host-directed adjunctive treatments like vitamin D supplementation are attractive; nevertheless, their therapeutic value is still unclear, especially in MDR-TB (Girishbhai Patel et al., 2024).

Crucially, research on drug-susceptible TB provides the majority of the data that currently exists connecting vitamin D status and VDR polymorphisms to TB outcomes. MDR-TB-specific data are few, usually underpowered, and typically extrapolated from diverse populations. Standardized outcome criteria have not been used to systematically assess the impact of vitamin D–VDR interactions on bacteriological conversion, treatment response, and prognosis in MDR-TB (Rathored et al., 2025).

In light of this, the current systematic review summarizes research on the relationship between vitamin D level, VDR genetic variants, and tuberculosis outcomes that was published between 2000 to 2024, with an emphasis on MDR-TB (Sekhar Miraj et al., 2022). This review attempts to elucidate the strength, limits, and translational significance of vitamin D–VDR pathways in MDR-TB care by segregating data across dimensions of susceptibility, treatment response, and prognosis and by matching outcomes with WHO criteria.

## Materials and methods

### Literature search strategy

A systematic review was conducted to evaluate the association of vitamin D receptor (VDR) genetic variants and therapeutic response in multidrug-resistant pulmonary tuberculosis (MDR-TB). Comprehensive searches of peer-reviewed literature were performed using databases including PubMed, Web of Science, and Google Scholar. The search covered publications from 2000 to 2024 using keywords: “tuberculosis”, “MDR-TB”, “pulmonary tuberculosis”, “vitamin D”, “VDR polymorphism”, “immune response”, and “treatment outcome”. Reference lists of included studies were scanned for additional relevant sources.

### Selection criteria

Inclusion criteria comprised: original research and review articles studying VDR gene polymorphisms (notably FokI, TaqI, BsmI, ApaI) or vitamin D status in association with TB susceptibility or outcomes; studies involving human populations with PTB or MDR-TB; English-language publications. Exclusion criteria were: case reports, animal-only studies, studies lacking clear data on VDR polymorphisms, or duplication of datasets.

### Data extraction

Two independent reviewers extracted data on study design, population demographics, sample size, VDR genotypes tested, vitamin D measurement methods, therapeutic response parameters, and relevant statistical findings. Discrepancies were resolved via consensus.

### Quality assessment

The quality of included studies was evaluated using established tools, such as the Newcastle-Ottawa Scale (NOS) for cohort and case-control designs, and Cochrane risk-of-bias tools for randomized trials. Review articles and meta-analyses were analyzed for methodological rigor and relevance. For NOS, domain scores were assigned for Selection (0–4), Comparability (0–2), and Outcome/Exposure (0–3), summed to a total (0–9) and interpreted as low (7–9), moderate (5–6), or high (0–4) risk of bias; brief domain justifications are provided alongside study−level entries.

For RoB 2, five domains were assessed as Low, Some Concerns, or High, and the overall risk of bias was derived according to the RoB 2 mapping scheme; judgments were supported by signalling-question evidence from each trial report or registry, where available.

### Data analysis

Data synthesis focused on pooled effect estimates for VDR polymorphism associations with TB susceptibility and treatment outcomes where available. Heterogeneity between studies was assessed qualitatively, with subgroup analyses by geographic region, ethnicity, and TB phenotype when data allowed.

Outcomes and synthesis were prespecified in three domains: (i) susceptibility to pulmonary TB (host genetic or vitamin D status associations with risk), (ii) MDR−TB treatment response (on−treatment bacteriological conversion and clinical dynamics), and (iii) prognosis (end−of−treatment outcomes per WHO), with extraction and synthesis conducted separately for each domain to enhance clarity and comparability in line with PRISMA 2020 reporting guidance. For treatment response, culture conversion was defined where reported as two consecutive negative cultures collected at least 28–30 days apart, consistent with widely used research definitions and WHO monitoring guidance, noting historical variation across studies in the exact interval used. Prognosis outcomes (cure, treatment completed, failure, death, loss to follow−up, and optional sustained success in operational research) followed the harmonized WHO 2020 definitions applied across DS−TB and DR−TB to support consistent outcome assignment and interpretation. Studies enrolling only drug−susceptible TB were excluded from MDR−TB response syntheses but could contribute to susceptibility or mechanistic context if prespecified, and all study−level data were mapped into a single table with three labeled sections capturing country, sample size, design, key results, and conclusions for transparent domain−specific appraisal.

### Ethical approval

As a systematic review of published literature, this study did not involve new data collection from human subjects and therefore did not require institutional ethics approval. Data privacy and consent provisions from original studies were respected throughout the review process.

## Results

A total of 213 studies from 2000 to 2024 were synthesized, categorized into three domains: susceptibility, treatment response, and prognosis, with references to supporting tables and figures for clarity and transparency.

### Study quality and risk of bias

**Tables 1** and **2** summarize the methodological quality and bias risk of the included research. One of the five case-control studies that were assessed using the Newcastle–Ottawa Scale (**Table 1**) had a low risk of bias, three had a moderate risk, and one had a high risk, mostly as a result of imprecise reporting of non-response and insufficient confounding correction. The majority of randomized controlled trials evaluated using the Cochrane RoB 2 technique (**Table 2**) were categorized as having “some concerns,” mostly because of unclear allocation concealment and protocol violations. These evaluations offer crucial background information for understanding the degree of correlations described in the sections that follow.

**Table 1:** Risk of bias summary for observational studies (Newcastle–Ottawa Scale).

Study (year, country)	Design	Selection (0–4)	Comparability (0–2)	Outcome/exposure (0–3)	Total (0–9)	Overall risk of bias	Remarks
Wilkinson et al., 2000 (Gujarati Asians, UK) (Wilkinson et al., 2000)	Case–control	4	1	2	7	Low	Clear case definition and community controls; genotype and vitamin D status measured uniformly in cases/controls; partial adjustment for baseline factors; non-response not detailed but exposure methods robust.
Roth et al., 2004 (Peru) (Roth et al., 2004)	Case–control	3	1	2	6	Moderate	Adequate case definition; controls reasonably selected; uniform genotyping; limited adjustment for confounders reported in summary; non-response handling unclear.
Babb et al., 2007 (South Africa) (Babb et al., 2007)	Case–control	2	0	2	4	High	Control selection and matching limited in summary; uniform exposure ascertainment via genotyping; no explicit confounder control described.
Zhang et al., 2017 (China; bone/joint TB) (Waldman, 1974)	Case–control	3	1	2	6	Moderate	Clinic-based cases with appropriate controls; uniform PCR-based genotyping; some adjustment indicated; non-response not detailed.
Rathored et al., 2012 (North India; MDR-TB, Cat I, controls) (Rathored et al., 2012)	Case–control (three-arm)	3	1	2	6	Moderate	Multi-group design with consistent exposure ascertainment; limited but present adjustment; response/non-response processes not fully specified.

Risk of bias in observational studies assessed with the Newcastle–Ottawa Scale (NOS), summarizing domain scores for Selection (0–4), Comparability (0–2), and Outcome/Exposure (0–3), total NOS score (0–9), and overall judgment (low 7–9, moderate 5–6, high 0–4) per established guidance.

**Table 2:** Risk of bias summary for randomized trials (Cochrane RoB 2).

Trial (year, country)	Randomization process	Deviations from intended interventions	Missing outcome data	Measurement of the outcome	Selection of the reported result	Overall risk of bias	Notes	
Martineau et al., 2011 (UK) vitamin D3 vs placebo, intensive phase PTB (Martineau et al., 2011)	Some concerns (sequence/allocation not detailed in manuscript excerpt)	Low (double-blind RCT reported)	Low (no major imbalance noted in manuscript excerpt)	Low (microbiological outcomes standardized)	Some concerns (protocol/reporting not described in manuscript excerpt)	Some concerns	Confirm from full text: registration/protocol, allocation concealment, domain justifications; manuscript excerpt indicates double-blind design and culture/sputum outcomes.
Wejse et al., 2009 (Guinea-Bissau) vitamin D adjunct vs placebo (Wejse et al., 2009)	Some concerns (details not in manuscript excerpt)	Low (double-blind, placebo-controlled)	Low (no major imbalance noted in manuscript excerpt)	Low (standard TB outcomes)	Some concerns (selective reporting not assessable from excerpt)	Some concerns	Verify registration, allocation concealment, and pre-specified outcomes from article; manuscript notes double-blind placebo trial.
Mily et al., 2015 (Bangladesh) PBA+vitamin D3 adjunct RCT (Mily et al., 2015)	Some concerns (randomization specifics not in excerpt)	Low (randomized adjunct design)	Low (no differential attrition noted in excerpt)	Low (clinical/microbiological outcomes)	Some concerns (protocol availability not in excerpt)	Some concerns	Check trial registry, outcome switching, and allocation concealment in full text for final judgments.

Risk of bias in randomized trials assessed with Cochrane RoB 2 across five domains (randomization process; deviations from intended interventions; missing outcome data; measurement of outcomes; selection of the reported result) with overall judgments mapped per RoB 2 rules (Low only if all domains Low; High if any domain High; otherwise Some concerns).

### Susceptibility, treatment response, and prognosis

*Susceptibility:***Table 3A** summarizes the evidence that links TB susceptibility to VDR polymorphisms. Significant correlations between particular VDR variants (most notably FokI, BsmI, TaqI, and ApaI) and susceptibility to pulmonary tuberculosis in specific groups have been found in trial-sequential meta-analyses and population-based research, suggesting a genetic component to disease risk. Nonetheless, a number of case-control studies revealed null relationships, emphasizing variability particular to a given group. The conclusion that VDR polymorphisms affect TB susceptibility in an ethnicity- and context-dependent way rather than serving as universal risk markers is supported by these conflicting results, as shown in **Table 3A**.

**Table 3:** Evidence on VDR polymorphisms, vitamin D status, and pulmonary TB outcomes organized by domain: susceptibility, treatment response, and prognosis.

A. Susceptibility (host genetic or metabolic factors associated with TB risk)					
Study (year; reference)	Country	Sample size	Design	Key result	Conclusion
Aparna P et al., 2024 (Aparna et al., 2018)	India	>5,000 (cumulative)	Trial sequential meta-analysis	VDR SNPs (FokI, BsmI, TaqI, ApaI) significantly associated with TB susceptibility	VDR variants influence TB susceptibility; precision risk stratification warranted
Wu Q et al., 2022 (Wu et al., 2022)	Worldwide	24 studies	Systematic review/meta-analysis	T2DM-PTB incidence 129.89/100,000 PY; prevalence 511.19/100,000	High PTB burden among people with T2DM; prevention priority in high-TB settings
Babb CL et al., 2007 (Babb et al., 2007)	South Africa	249 cases; 352 controls	Case–control	No VDR genotype association with TB susceptibility in admixed population	Null susceptibility association despite functional plausibility
Roth DE et al., 2004 (Roth et al., 2004)	Peru	103 cases; 206 controls	Case–control	No significant VDR association with susceptibility	Null in case–control; see cohort phase for treatment response
B. Treatment response (on-treatment bacteriological conversion and clinical dynamics)					
Study (year; reference)	Country	Sample size	Design	Key result (conversion criteria where stated)	Conclusion
Rathored et al., 2024 (Rathored et al., 2024)	India	897 (Cat I + MDR-TB)	Programmatic cohort	PTB Cat I mean smear 0.95 ± 0.7 mo, culture 0.8 ± 0.7 mo; MDR-TB smear 2.4 ± 3 mo	Earlier conversion predicts better prognosis; 2-month conversion marker for MDR-TB
Tiwari S et al., 2012 (Tiwari et al., 2012)	India	60 Cat I; 236 MDR-TB	Prospective cohort	PTB Cat I: BsmI Bb faster smear conversion than bb; MDR-TB: smear time negatively correlated with vitamin D	Genotype and vitamin D linked to on-treatment bacteriology
Martineau AR et al., 2011 (Martineau et al., 2011)	UK	126	RCT double-blind	Culture conversion 36 vs 43.5 d (HR 1.39, p=0.14); TaqI tt genotype modified effect (HR 8.09)	Vitamin D adjunct accelerated conversion in TaqI tt; no overall effect
Wejse C et al., 2009 (Wejse et al., 2009)	Guinea-Bissau	365	RCT double-blind	No difference in TB score or smear conversion rates with vitamin D	No overall clinical benefit from vitamin D adjunct in this trial
Roth DE et al., 2004 (Roth et al., 2004)	Peru	Cohort arm	Cohort	Faster smear/culture conversion with FokI FF and TaqI Tt genotypes	VDR genotypes associated with conversion speed during treatment
Babb CL et al., 2007 (Babb et al., 2007)	South Africa	222 cases	Cohort	VDR genotype predicted fast (<2 mo) vs slow (≥2 mo) responders (conversion to negativity)	Genetic variation relates to on-therapy conversion
Hibma JE et al., 2017 (Hibma et al., 2020)	Multinational	405 RPT; 252 RIF	PK-PD cohort	Time to culture conversion correlated with geography, rifapentine exposure, large cavity; lower exposure in HIV+/Black/male/fasting	RPT 1,200 mg/d recommended; large cavities attenuate response
Griffith DE et al., 2013 (Griffith et al., 2015)	Multinational	1,978	Retrospective pooled	Productive cough linked to higher baseline burden, slower resolution, greater culture positivity during treatment	Symptoms track microbiological response and may serve as pragmatic markers
C. Prognosis (end-of-treatment and post-treatment outcomes per WHO definitions)					
Study (year; reference)	Country	Sample size	Design	Key result (WHO outcome metrics)	Conclusion
Conradie F et al., 2020 (Conradie et al., 2020)	South Africa	109 highly DR-TB	BPaL cohort	90% favorable outcome at 6 mo post-treatment; toxicities: neuropathy, myelosuppression	BPaL regimen high favorable outcomes; manageable toxicity profile
Chaves-Torres et al., 2019 ((PDF) Factors predictive of the success of tuberculosis treatment: A systematic review with meta-analysis)	Global	151 studies	Systematic review	DS adult treatment success 80.1%; lower with HIV or MDR/XDR; multiple patient factors linked to success	Global success below 85% target; tailored interventions required
Imperial MZ et al., 2018 (Imperial et al., 2018)	Worldwide	3,411 (3 trials)	Individual participant pooled	HIV+, adherence ≤90%, smear 3+ predicted adverse outcomes; low smear/no cavitation easier-to-treat phenotype	Baseline burden and adherence stratify prognosis and regimen suitability
Leung CC et al., 2015 (Immigrants and tuberculosis in Hong Kong)	Hong Kong	16,345	Follow-up cohort	Smoking associated with extensive disease, lower cure/completion, higher relapse ≤2 yr	Smoking cessation may improve outcomes and reduce transmission
Legesse T et al., 2021 (Legesse et al., 2021)	Ethiopia	1,553 (2014–2017)	Programmatic surveillance	Rising notifications; higher childhood/EPTB; success below targets	Strengthen adherence education/supervision to raise success
Varughese M et al., 2023 (Varughese et al., 2023)	Indigenous global	24 studies	Review	Time to treatment 24–240 d; patient delay 20 d–2.5 yr; longer in Indigenous ≥60% studies	Access delays likely worsen prognosis; programmatic action needed
Velayutham BRV et al., 2014 (Velayutham et al., 2014)	South India	865 elderly (≥60 yr); 4,343 younger (15–59 yr)	Cohort	Elderly: more male, smokers, illiterate, smear-negative; higher LTFU and death; more side effects	Distinct profiles and outcomes in elderly; tailored management required
Kearns MD et al., 2014 (Kearns and Tangpricha, 2014)	Global	Review	Systematic review	Vitamin D enhances macrophage activity and suppresses *M. tuberculosis* proliferation	Vitamin D may serve as adjunct for immune response in TB
Imperial MZ et al., 2018 (Imperial et al., 2018)	Multinational	>12,000 participants	Patient-level pooled meta-analysis	Identified potential regimens for shortening TB treatment without compromising efficacy	Shortened regimens effective; may enhance adherence, reduce adverse events

Evidence on VDR polymorphisms, vitamin D status, and pulmonary TB outcomes organized by domain: susceptibility, treatment response, and prognosis. A. Susceptibility synthesizes host genetic and metabolic factors associated with TB risk in case–control or cohort designs, distinct from treatment outcomes. B. Treatment response covers on-treatment bacteriological dynamics (time to smear/culture conversion; proportion culture-negative at fixed time points) and clinical response during therapy; culture conversion defined as two consecutive negative cultures ≥30 days apart per WHO criteria. C. Prognosis summarizes end-of-treatment outcomes (cure, treatment completed, failure, death, loss to follow-up) and post-treatment relapse-free cure per WHO Module 4/operational handbook definitions. Studies may contribute to more than one domain if they report multiple outcome types; abbreviations are expanded below; references denoted in parentheses correspond to manuscript bibliography.

BPaL, bedaquiline + pretomanid + linezolid; BPaLM, bedaquiline + pretomanid + linezolid + moxifloxacin; Cat I, Category I (first-line drug-susceptible TB treatment regimen); CI, confidence interval; d, days; DR-TB, drug-resistant tuberculosis; DS, drug-susceptible; EPTB, extrapulmonary tuberculosis; HIV, human immunodeficiency virus; HR, hazard ratio; IQR, interquartile range; LTFU, loss to follow-up; MDR-TB, multidrug-resistant tuberculosis; mo, months; *M. tuberculosis*, *Mycobacterium tuberculosis*; OR, odds ratio; PK-PD, pharmacokinetic–pharmacodynamic; PTB, pulmonary tuberculosis; PY, person-years; RCT, randomized controlled trial; RIF, rifampicin; RPT, rifapentine; SNP, single nucleotide polymorphism; T2DM, type 2 diabetes mellitus; TB, tuberculosis; VDR, vitamin D receptor; WHO, World Health Organization; XDR, extensively drug-resistant tuberculosis; yr, years. ¶ Listed twice for distinct outcome domains

The majority of the research listed in **Table 3A** are candidate-gene studies carried out in drug-susceptible or general TB populations. These findings do not particularly address genetic determinants of multidrug-resistant TB, but they do offer valuable Figureinsights into host genetic vulnerability to TB, including the impact of VDR polymorphisms and metabolic comorbidities. Therefore, rather than indicating risk unique to resistance, the findings derived from this data should be viewed as reflecting general TB susceptibility. In order to enhance risk stratification and treatment accuracy, future research is necessary to explicitly assess genetic correlations in MDR-TB populations.

*Treatment response:* The data on treatment response shows a considerable difference between drug-susceptible TB (DS-TB) and multidrug-resistant TB (MDR-TB) groups, as shown in **Table 3B** and **s 2**, **3**. Although they were not taken as direct evidence for MDR-TB treatment results, studies carried out solely in DS-TB populations were included in **Table 3B** to offer contextual and mechanistic insight into vitamin D–VDR–mediated effects on bacteriological conversion.

As shown in **Table 3B**, MDR-TB-specific results in our review are consequently exclusively drawn from trials that specifically included MDR-TB patients. These investigations show that any correlations between vitamin D status or VDR polymorphisms and culture or smear conversion are weak and genotype-dependent rather than consistent, and that treatment response in MDR-TB is significantly slower and more diverse than in DS-TB.

**Figure 1:**
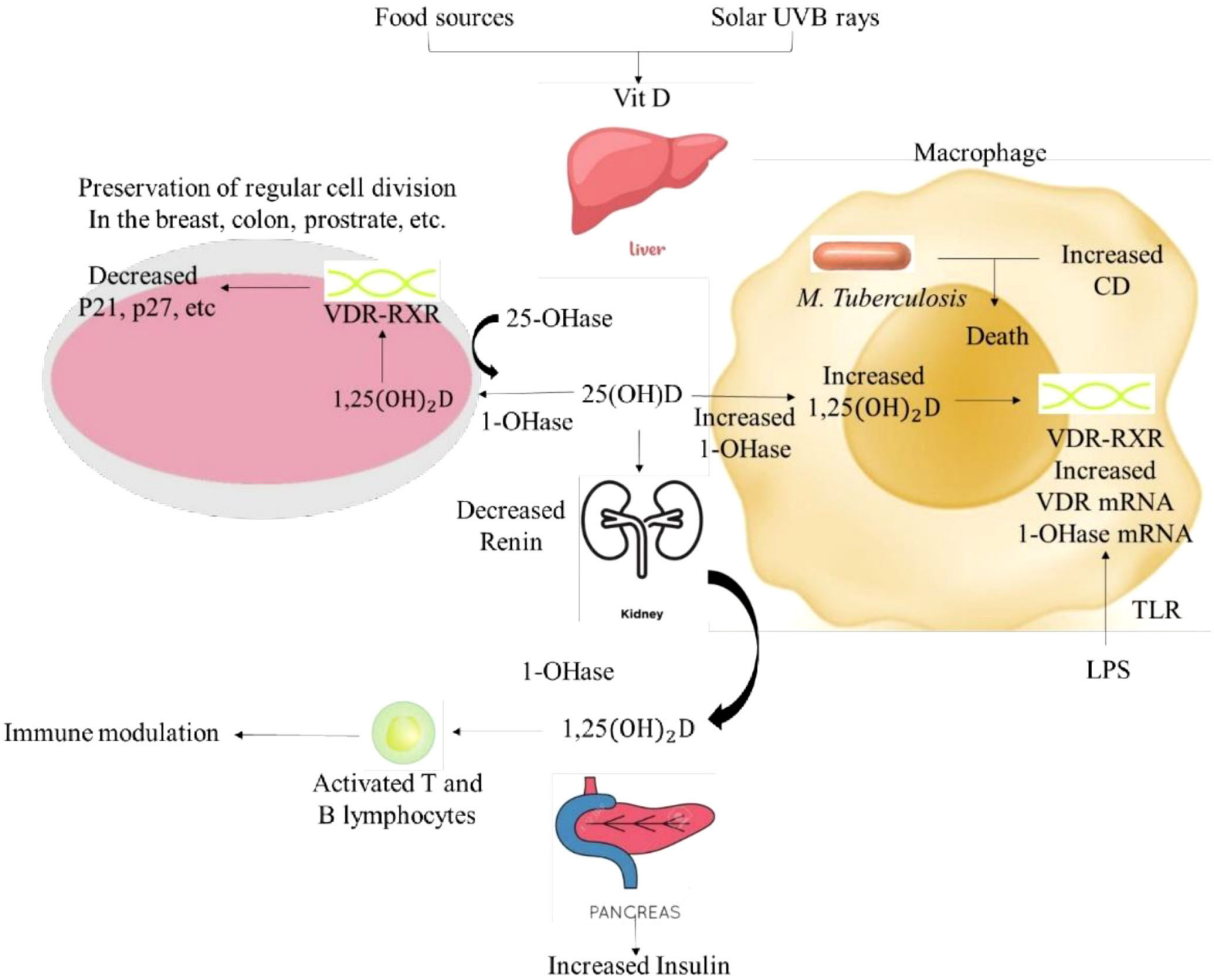
Role and importance of vitamin D in human body. The figure depicts the vitamin D–VDR pathway and induction of antimicrobial peptide cathelicidin, illustrating a mechanistic link to mycobacterial killing and immune modulation relevant to TB pathogenesis. Vit D, vitamin D; 25-OHase, 25-hydroxylase; 1-OHase, 1-alpha-hydroxylase; 25(OH)D, 25-hydroxyvitamin D; 1,25(OH)_2_D, 1,25-dihydroxyvitamin D; VDR, Vitamin D Receptor; RXR, retinoid X receptor; CD, cathelicidin (antimicrobial peptide); TLR, toll-like receptor; LPS, lipopolysaccharide; mRNA, messenger RNA; P21, cyclin-dependent kinase inhibitor 21; P27, cyclin-dependent kinase inhibitor 27.

**Figure 2:**
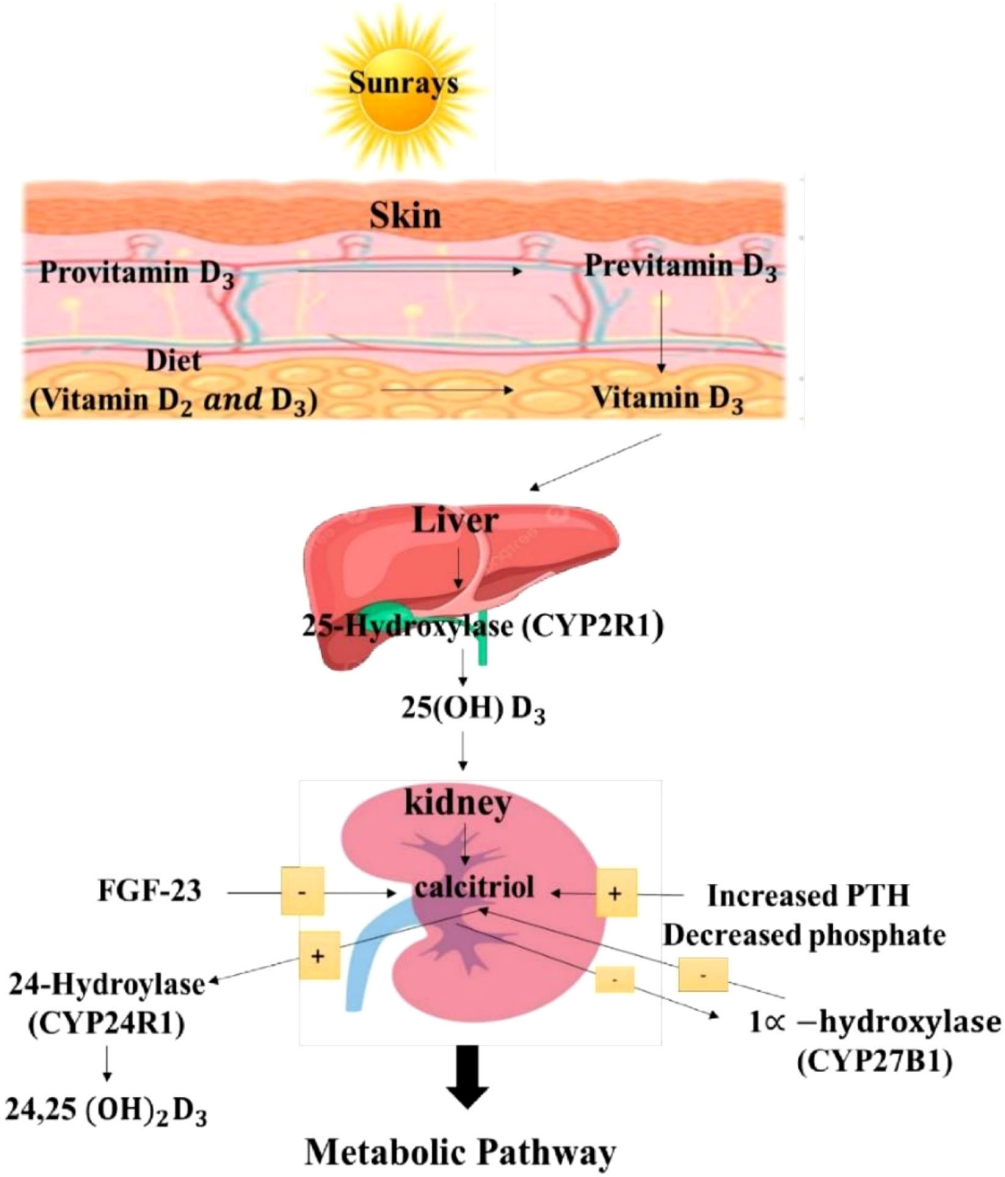
Metabolic function of vitamin D in human body. Vitamin D metabolism in humans: UVB-driven conversion of cutaneous provitamin D3 to previtamin D3 and vitamin D3 with dietary vitamin D2/D3 input, hepatic 25−hydroxylation by CYP2R1 to 25(OH)D3, renal 1α−hydroxylation by CYP27B1 to calcitriol, and catabolism via CYP24R1 to 24,25(OH)2D3. Renal activation is stimulated by increased PTH and decreased phosphate and inhibited by FGF−23, illustrating endocrine feedback regulation of 1α−hydroxylase and 24−hydroxylase activity. Vit D3, vitamin D3; Vit D2, Vitamin D2; Provit D3, provitamin D3; Previt D3, previtamin D3; CYP2R1, cytochrome P450 2R1 (25-hydroxylase enzyme); 25(OH)D3, 25-hydroxyvitamin D3; CYP27B1, cytochrome P450 27B1 (1α-hydroxylase enzyme); Calcitriol, 1,25-dihydroxyvitamin D3 (active form of Vitamin D); FGF-23, fibroblast growth factor 23; PTH, parathyroid hormone; CYP24R1, cytochrome P450 24R1 (24-hydroxylase enzyme); 24,25(OH)_2_D3, 24,25-dihydroxyvitamin D3.

While no consistent overall effect was shown across all MDR-TB patients, vitamin D supplementation or favorable VDR genotypes were linked to rapid conversion in certain genetic groupings, as shown in **Figures 1**, **2**. Crucially, rather than bolstering extrapolative results, DS-TB studies were solely utilized to compare conversion kinetics and biological plausibility, emphasizing the unique treatment setting of MDR-TB.

Accordingly, this review does not generalize DS-TB findings to MDR-TB but instead uses them to emphasize that host-directed effects may be attenuated, context-specific, and modified by resistance profile, as reflected in the structured separation of evidence within **Table 3B**.

*Prognosis:* A range of endpoints from both drug-susceptible tuberculosis (DS-TB) and multidrug-resistant tuberculosis (MDR-TB) cohorts are represented by the prognostic outcomes listed in **Table 3C**, which reflects variation in research design, treatment plans, and outcome definitions. When results were provided, they were mapped to harmonized WHO end-of-treatment categories to guarantee interpretability. This allowed for descriptive cross-study comparison without suggesting a direct equivalency between DS-TB and MDR-TB patients.

Studies of modern MDR-TB regimens reveal generally positive treatment success rates, as **Table 3C** illustrates, but they also consistently highlight programmatic and clinical predictors of worse outcomes, such as high baseline disease load, delayed diagnosis, and concomitant illnesses. Crucially, host genetic variables and vitamin D status were interpreted in connection with treatment context and standardized outcome categories rather from being assessed as independent predictive predictors.

**Figure 3:**
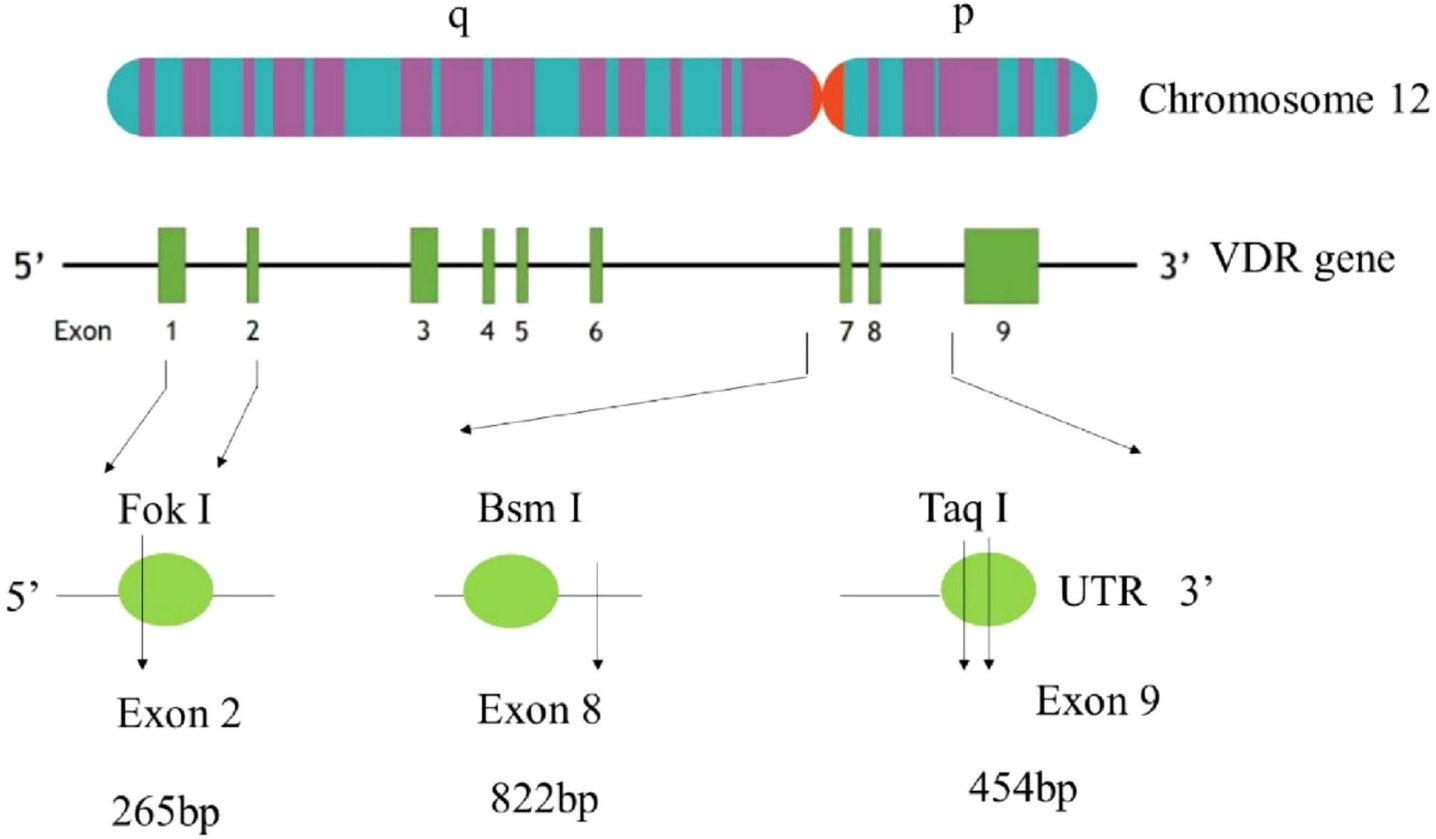
Gene arrangements and restriction sites of the vitamin D receptor (VDR) gene. Genomic organization of the human VDR gene on chromosome 12q13 showing exons 1–9 and the positions of commonly studied restriction polymorphisms: FokI at the translation start site in exon 2 (rs2228570), BsmI in intron 8 near the 3′ end (rs1544410), and TaqI in exon 9/3′ UTR (rs731236). PCR–RFLP genotyping at these loci uses enzyme−specific fragment patterns to infer alleles, as illustrated by the representative amplicon sizes shown in the schematic. VDR, vitamin D receptor; UTR, untranslated region.

An integrated conceptual framework that shows how host vulnerability, on-treatment bacteriological response, and ultimate prognosis are related but analytically separate domains is presented in **Figure 3**. This concept highlights that rather from being regarded as separate genetic or dietary effects, prognostic results in MDR-TB should be viewed within a multidimensional environment formed by host characteristics, treatment regimens, and programmatic variables.

### Integrated interpretation of results

**Figure 4:**
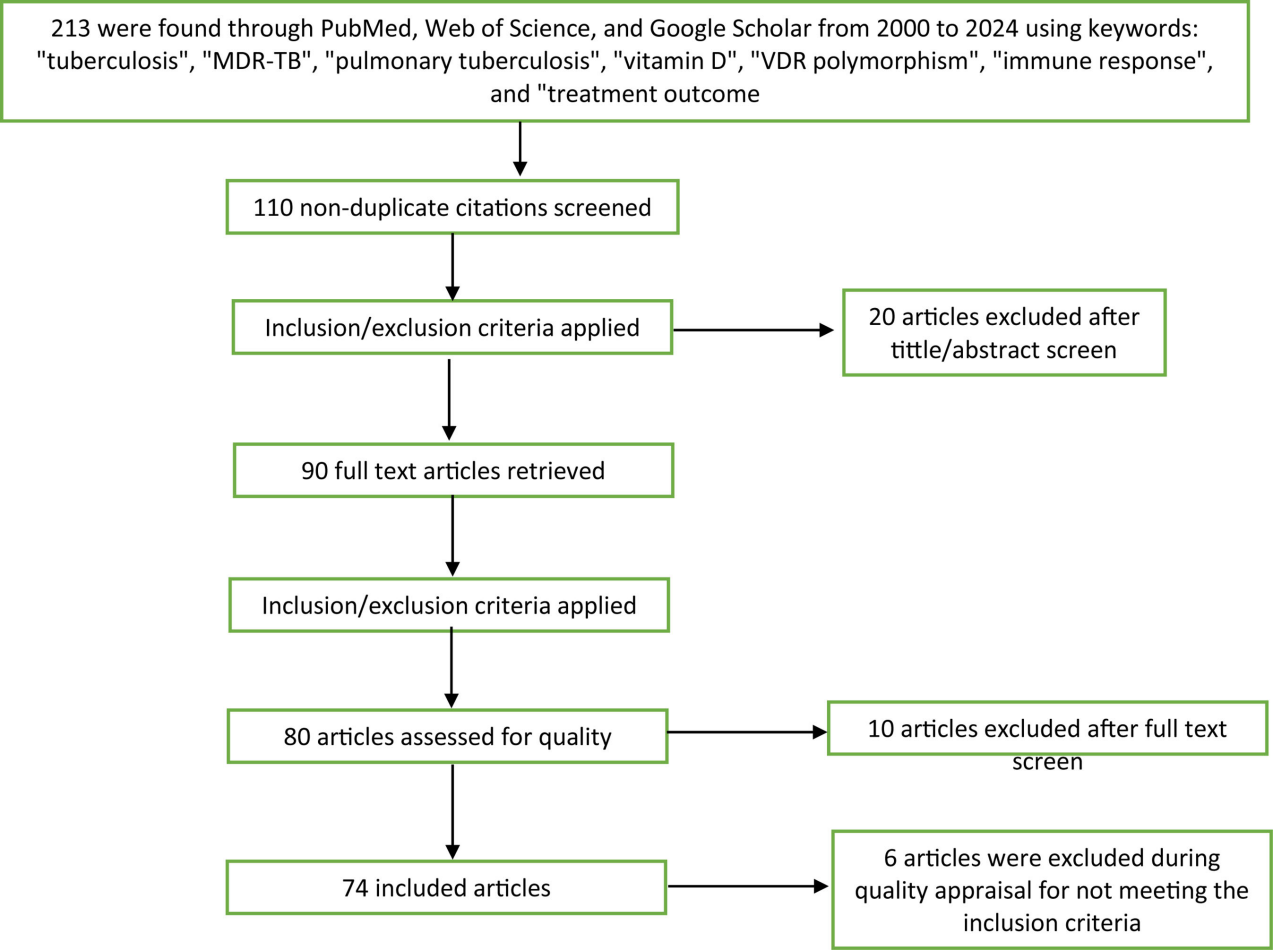
Flow diagram for new systematic review on VDR/vitamin D and MDR−PTB outcomes.

Together, **Tables 3A–C** and **Figures 3**, **4** offer an organized framework that connects ultimate prognosis, on-treatment dynamics, and host susceptibility. This domain-specific organization reduces narrative ambiguity and strengthens the evidence supporting the conclusions reached by elucidating how vitamin D status and VDR polymorphisms relate to TB risk, impact bacteriological response during therapy, and ultimately affect treatment outcomes.

### Immune response to *Mycobacterium tuberculosis* infection

The host defense system against *Mycobacterium tuberculosis* infection integrates both innate immunity (natural resistance) and acquired (adaptive) immunity. Macrophages and cytotoxic lymphocytes (NK cells, CD8+ T cells, and CD4+ T cells) are components of innate immunity, which consists of preventing the growth and directly lysing *Mycobacterium tuberculosis*. Phagocytosis of *Mycobacterium tuberculosis* is mediated by several cell surface molecules on the macrophage, which bind either to nonopsonized *Mycobacterium tuberculosis* or recognize opsonins on the surface of the *Mycobacterium tuberculosis* (Chai et al., 2019). Toll-like receptors (TLRs), which are essential for innate immunity, are responsible for the recognition of Mycobacterium tuberculosis. *Mycobacterium tuberculosis* can be killed or its growth inhibited, as well as the process intensified, by the cytokines produced when cells are activated by recognition. The immune system’s responses may lead to defense against the invasive infection. These reactions, however, have the potential to harm the host and benefit the pathogen if they are excessive or improper. There may be a balance between the different reactions; this is dependent on both natural and genetic variables (Duan et al., 2022). One of the most important responses that should be in balance is between the two phenotypes of the T-helper cell, Th1 and Th2, and the cytokines they release. Th1 facilitates a protective cell-mediated response, while Th2 facilitates antibody formation and inhibits the production of IFN-γ and activation of the macrophage, and consequently weakens the immune system. A pure Th1 cytokine response (high levels of IL-12 and IFNγ expression) facilitates protection, but a mixed Th1/Th2 response leads to tissue-destroying hypersensitivity and progression to disease. However, the Th1/Th2 conception in disease vulnerability remains tentative (Supriya et al., 2021).

### Nutrition and diet

Malnutrition is a global concern, significantly compromising immune function and exacerbating immunodeficiency. From hindering the effectiveness of innate and acquired immunity to impairing cell-mediated responses crucial in combating diseases like TB, its impact intensifies with the severity of malnourishment, underscoring the critical interplay between nutrition and immune health on a global scale. Micronutrients also occupy a crucial role in the functioning of immunity; besides this, their deficiencies can elevate the susceptibility to infection (Morales et al., 2023).

Studies, including one in India, indicate that an improved diet during treatment not only leads to weight gain but also hastens recovery, evident in accelerated smear conversion and resolution of radiographic abnormalities. These findings extend to MDR-TB cases, highlighting the pronounced association between malnutrition severity and clinical symptom severity in such patients. Several researchers propose that the deficiency of micronutrients and trace elements is linked with the development of TB, as their supplementation may improve the T cell count and reduce complications, and may help in gaining weight (Gler et al., 2013).

Though abundant in many parts of the world where sunlight is abundant year-round, vitamin D deficiency is a common issue. The body needs food and sunlight for the synthesis of vitamin D because most foods are naturally high in animal derived vitamin D (Dominguez et al., 2021). Apart from dietary advice, the solar zenith angle which is impacted by latitude and time of day is an important element in UVB light absorption and affects the skin’s capacity to generate vitamin D (Harinarayan et al., 2013). Furthermore, the use of sunscreen and outdoor clothing choices further modulates UV exposure levels. The amount of UVB radiation absorbed depends on the length of time spent in the sun and the body surface area (BSA) exposed. Although the nutrient requirement for MDR-TB patients has not been proposed so far but nutrient prerequisites in TB have been recommended. It is considered that during active TB, nutrient requirement rises due to a hyper-catabolic state by approximately 35–40 kcal per kg of ideal body weight (Lu and Ilyas, [[NoYear]]). Protein requirement furthermore increases due to considerable wasting of body tissues during disease and is thought to be about 1.2 to 1.5g of protein/kg/day or 15% of total energy intake, i.e., 75-100g approximately daily. Also, Low blood levels of trace elements like zinc, selenium, and iron in TB patients necessitate an augmented dietary intake, while vitamin B6 supplementation is crucial due to isoniazid-induced deficiency (Lonnie et al., 2018). Furthermore, the link between TB and vitamin D deficiency underscores its potential role in modulating immune function against Mycobacterium tuberculosis, historically supplemented by sunlight exposure and fish liver oils before antimicrobial therapies emerged (Kearns and Tangpricha, 2014).

### Population component

There is compelling evidence that an individual’s susceptibility to Mycobacterium TB infection and the subsequent development of clinical illness is significantly influenced by their genetic composition (Möller et al., 2018). Compared to groups (Europeans) in the mature stage of the epidemic that have evolved intrinsic resistance, a population that has just been exposed to an infectious disease is typically more vulnerable since there is less selection pressure. Inter-population and racial differences in susceptibility to TB have been observed (Möller et al., 2018). Various studies on racial variation in susceptibility to TB indicate that black people are twice as likely to develop TB as white individuals, even when exposed to the same social conditions. In the United States, American Indians, Black people, and Yupik are highly susceptible to TB, while resistance is observed in Caucasian and Jewish populations. Conducted a study of tuberculin conversion following exposure to infection in residents of Arkansas nursing homes and found that Black people have higher evidence for new infection (taking into account socioeconomic variables). Conversely, a variation in the frequency of disease between these groups was not established (Nahid et al., 2011).

### Linkage studies

A linkage study comprises family-based and involves scanning the entire genome to find all genes that exert a major effect on susceptibility to TB. Moderate effects are frequently/often missed due to the low power of this technique. This study recruits large numbers of families that have two or more affected sibling pairs. The study determines if a highly polymorphic marker is inherited by the offspring, it determines the number of alleles shared by the siblings. Once linkage has identified a region segregating with disease, association with candidate markers/gene polymorphisms can be used in the fine mapping of the region to localize the disease susceptibility gene (Aravindan, 2019).

### Association studies

Candidate gene analysis is used to identify genes that exert a small or moderate effect on susceptibility to infection, and generally has a higher power than linkage studies. However, there are several limitations to this technique. This approach is limited to known candidate genes and, therefore, genes with major effect but unknown function may be excluded/missed. If a positive result is achieved, an appropriate correction for multiple comparisons is considered prudent. However, when correcting for multiple alleles, a lack of power can result in only extremely strong associations being demonstrated. Another problem linked with the candidate gene approach is population admixture (Amos et al., 2011). Association can exist due to population stratification. To overcome the problem of ethnically heterogeneous populations, a family-based approach can be used. This approach recruits the affected individual as a case and his/her parents as internal controls. Significant differences between cases and controls can either be due to chance, a confounding factor, or the variant itself being a disease-susceptible allele (Lin and Chen, 2010). The allele may be directly associated with the disease or could have no direct effect on the phenotype, but is in linkage disequilibrium (LD) with another allele associated with the disease. LD is due to an occurrence of two alleles of two genes, which form a haplotype that occurs at frequencies higher than expected for the frequencies of the single alleles (Hayeck et al., 2024). Distribution of alleles can be observed in the Hardy-Weinburg equilibrium. In addition to genotyping errors, the Hardy-Weinberg equilibrium may deviate due to non-random mating, migration into or out of the population, multiple genotype survivorships, population stratification, admixture of different ethnic groups, and stochastic fluctuations caused by small samples (Chen et al., 2017). Therefore, choosing adequate controls is essential in complementary/association research, but it can be difficult due to unknown confounding brought on by variations in ancestry, ethnicity, or mixing between cases and controls.

### Case-control studies

Case-control (association) studies involve comparison of the frequency of variant alleles at a candidate locus between affected individuals and matched unrelated, unaffected controls. In broad-spectrum studies, a large sample size is required to investigate the role of rare or multiallelic genes in susceptibility to disease. The selection of candidate genes is based on their function, probable significance for the disease under consideration, or the presence of one or more genetic variants. Studies in mice, including gene knockout mice, have identified several candidate genes involved in disease susceptibility to infectious disease (Clarke et al., 2011).

### Vitamin D receptor

#### Biology of vitamin D

The steroid receptor family consists of members of the VDR. Vitamin D coexists in two forms: Vitamin D_2_ (ergocalciferol) and vitamin D_3_ (cholecalciferol). Vitamin D_3_ is derived from the photolytic cleavage of the B rings of 7-dehydrocholesterol in the skin by exposure to ultraviolet light, in the presence of the sun. Nutritional resources of vitamin D include egg yolk, fish oil, liver, and a number of vegetables. High consumption of whole grain cereal products can impair bone metabolism by limiting calcium intake, which in turn affects the metabolism of vitamin D (Bikle, 2000). Human studies have demonstrated that consuming large amounts of whole grain cereal can improve the intestinal lumen’s ability to eliminate 25(OH) D3. Vitamins D2 and D3 are not hormone-active; they only become biologically active through metabolic processes. This includes the 1α-hydroxylation in the kidney and the 25-hydroxylation in the liver. Vitamin D, whether from the diet or from the photosynthesis of previtamin D, is converted into 25(OH)D by the liver’s vitamin 25-OHase. 1. OH, in the kidneys, ase transforms 25(OH)D (Liu et al., 2025). In addition to controlling the metabolism of calcium and phosphorus, 1,25(OH)2D can also induce the pancreas to produce insulin and inhibit the kidneys’ ability to produce renin. In addition, 1,25(OH)2D interacts with its nuclear receptor (VDR) in a range of tissues and cells to support healthy cell division and proliferation. Many different types of cells, such as those found in the breast, prostate, and colon, can also convert 25(OH)D to 1,25(OH)2D for autocrine production of 1,25(OH)2D. It is thought that controlling cell growth and maturation, which lowers the likelihood of the cell developing into a malignant one, is facilitated by the autocrine synthesis of 1,25(OH)2D (Bikle, 2014). In macrophages, 1-OHase metabolizes 25(OH)D to produce 1,25(OH)2D. When LPS stimulates TLR2/1, it upregulates the expression of the VDR and 1-OHase. The VDR and 1-OHase express themselves more as a result of this. The nuclear expression of cathelicidin (CD), a cationic peptide that destroys infectious agents like *Mycobacterium tuberculosis*, is increased in macrophages when 1,25(OH)2D production increases (**Figure 4**).

#### Synthesis and metabolism of vitamin D

The skin produces vitamin D first by nonenzymatically converting provitamin D3 to previtamin D3. The form of vitamin D that circulates in the highest concentration and is a reflection of exposure to sunlight and diet is 25(OH) D3, which is either converted in the liver by the enzyme or stored in adipose tissue. Supplements are commonly used, although there are few foods, aside from certain types of fish, that contain significant amounts of vitamin D. While 1α-hydroxylase is active in other tissues, it is converted in the kidney to the active metabolite 1,25(OH)2D, or calcitriol (Uçar and Holick, 2025). PTH levels rise in response to decreased serum calcium levels, which in turn promotes the synthesis of calcitriol. The synthesis of calcitriol can also be increased by lower serum phosphate levels. Fibroblast growth factor 23 (FGF-23), which is secreted by osteocytes in the bone matrix, inhibits its synthesis. A vitamin D product that is not biologically active, 25(OH)2 D3, is produced when calcitriol stimulates the activity of 24-hydroxylase (CYP24R1) and inhibits the activity of 1α-hydroxylase (CYP27B1) as seen in **Figure 1**, CYP stands for cytochrome P in CYP2R1, CYP27B1, and CYP24R1 (Bergwitz and Jüppner, 2010).

### Regulation of the vitamin D 25-hydroxylases

According to earlier studies, calcitriol therapy inhibits the rat liver microsomes’ capacity to perform 25-hydroxylation. Additionally, calcitriol has been shown to downregulate CYP27A1 mRNA in rats and the fetal gut. 1,25 (OH)_2_ vitamin D_3_ is the main regulator of calcium and phosphorus levels in serum and tissues. Initially in the liver and then in the kidney, vitamin D_3_ undergoes a two-step hydroxylation to form the active metabolite 1,25(OH)_2_D_3_ (Theodoropoulos et al., 2003). The hormone first operates via an intracellular receptor protein to alter the transcription of particular genes in target tissues. Furthermore, to comprehend the physiological reactions to vitamin D, one must be aware of the 1,25(OH)2D3 receptor’s regulation process. The findings that the biological response to 1,25(OH)2D3 is a direct function of receptor number and occupancy highlight the significance of receptor modulation (Voltan et al., 2023). **Figure 1**.

### Vitamin D mode of action

#### Nuclear receptors

Gene transcription is one way that vitamin D influences the body and causes its different effects. Both calcitriol and calcidiol are ligands for the VDR; however, calcidiol has a far lower affinity between 1/600 and 1/700^th^ than calcitriol does. The family of steroid nuclear receptors includes VDR. It functions as a transcription factor in a heterodimer with the retinoid X receptor (RXR). Vitamin D response elements (VDREs) are specific DNA sequences that are recognized by the VDR-RXR complex. A hexameric direct repeat of 5’-RGKTCA-3′, with R=A or G and K=G or T, is the consensus sequence of a VDRE. The two hexameric halfsites are divided by a three-nucleotide spacer. In the VDRE, the VDR-RXR complex takes up specific space: VDR binds to the 3′ halfsite while RXR binds to the 5’ halfsite; however, there are also reports that RXR binds to the 3′ halfsite (Bikle, 2000). The transcription of a gene can either activate or repress the active forms of vitamin D. When ligands bind to the VDR, transcriptional activation results in a conformational shift of the protein. This makes the portions of the VDR that can bind coactivator proteins visible. These, in turn, have the ability to either recruit other proteins that are also capable of histone acetylation or to remodel chromatin through histone acetylation themselves. Their actions allow transcription by exposing and opening up the DNA. Additionally, vitamin D can inhibit gene transcription. Corepressors are activated upon the binding of the VDR-RXR-complex to a negative VDRE. Either the corepressors recruit histone deacetylases or they possess histone deacetylation properties. Histone deacetylation inhibits transcription and chromatin exposure (Yao et al., 2024).

#### Molecular physiology of VDR

Hormonally dormant, vitamin D3 becomes biologically active when it undergoes metabolic processing. The intracellular pathogen *Mycobacterium tuberculosis* is found in macrophages. The idea that vitamin D acts as an immune system modulator is supported by the identification of VDRs on activated T, B, and monocytes. The active form of vitamin D, 1,25(OH)2D3, mediates regulation by binding to the VDR and performing its immunoregulatory function. Vitamin D maintains the balance between Th1 and Th2 cells. It accomplishes this through either positively or negatively regulating Th2 or Th1 cells. Some have proposed that vitamin D may be involved in the development or operation of Th cells because VDRs are present on Th cells. The precursor of Th1 is Th0; this precursor develops into Th1 once IFN-γ and IL-12 is secreted by monocytes or macrophages. 1,25(OH)_2_D_3_ decreases the production of Th1 and TH0 and increases the production of Th2 cytokines (Bishop E et al., 2021). *In vitro* studies have shown that incubation of active 1,25(OH)_2_D_3_ with human monocytes (which have VDR protein) results in an increased ability to inhibit the growth of *Mycobacterium tuberculosis*. Extrarenal production of 1,25(OH)2D3 at granuloma sites or in peripheral blood by activated T lymphocytes may enhance the macrophage’s capacity to suppress mycobacterium growth in TB patients (Weeres et al., 2014). On the other hand, a person with a vitamin D deficiency is more likely to develop TB due to a diminished monocyte-macrophage function **Figure 4**.

### Vitamin D and the immune system

The discovery that VDR is expressed in immune system cells suggested that vitamin D plays a role in the regulation of immune response. T cells, dendritic cells, monocytes, and macrophages all express VDRs. Even CYP27B1 and CYP24A1 are expressed by macrophages, suggesting that vitamin D plays a critical role in their operations. It’s interesting to note that the regulation of CYP27B1 in macrophages differs from that of the renal form of the enzyme; for example, 1,25(OH)2 D does not feedback regulate it. The function and maturation of T-lymphocytes on several levels are affected by the vitamin D levels. Unaware Th0 cells can differentiate into Th1 or Th2 cells based on the cytokines and activation method they are exposed to. Th1 and Th2 secrete distinct cytokines and can inhibit one another through distinct cytokine production. T-cell precursors (Th0) are presented with antigens by antigen-presenting cells (APCs) once they have been processed and bound to their major histocompatibility complex class II (MHC II) (Ghaseminejad-Raeini et al., 2023). Physical contact is established between the APC and Th0 when the MHC II on the APC combines with the T cell receptor on the Th0. Although insufficient for complete activation, this marks the start of the T cell precursor’s activation. Contact between Th0 and APC is facilitated by the interaction of proteins on the APC surface (such as CD80, CD86, and CD40) and those on the surface of the T-cell (such as CD28 and CD40L). APCs also release cytokines that promote Th1 development, such as IL-2 and IL-12. Type 1 cytokines (e.g., IL-2, INF-γ, and TNF-α) are stimulated to be expressed by IL-12, further inducing Th1 development.

One type of antigen-presenting cell that plays a significant role in the regulation of the immune system. Mature dendritic cells secrete IL-12, among other things, which stimulates Th0-cells to become Th1 cells. Th1-cells are linked to organ rejection and proinflammatory responses. Immature dendritic cells support tolerance. An increased Th1 activity is frequently observed in relation to autoimmune disorders. Conversely, Th2 cells exhibit greater regulatory capabilities. Cytokines such as IL-4, IL-5, IL-6, IL-9, IL-10, and IL-13 are secreted by Th2, and IL-4 specifically inhibits Th1 development and promotes Th2 formation. Th2-cell maturation is favored and Th1-cell formation is suppressed by vitamin D. As a result, the immune system becomes less reactive and tolerance levels rise. There are multiple steps involved in this. Vitamin D prevents the cytokine IL-12 from being produced, which promotes Th1 maturation and inhibits the formation of Th2. Additionally, vitamin D prevents MHC class II and surface receptors (such as CD40, CD80, and CD86) from being expressed on APC and DC, which reduces the likelihood that these cells will activate Th1 cells. Additionally, vitamin D increases the expression of IL-10, a cytokine that counteracts IL-12’s effects to further reduce the generation of Th1 cells.

### Deficiency of vitamin D and tuberculosis

Similar to TB, vitamin D deficiency is a serious public health issue in India. Before the development of antimicrobial drug therapy, there had been speculation about a connection between TB and vitamin D deficiency. Traditionally, TB was treated with fish liver oils and sunshine on occasion. It has been noted that a higher chance of developing active TB is associated with lower serum vitamin D levels. 1, 25(OH)2D plays a role in mineral and skeletal homeostasis and controls the growth and function of a wide range of cells, including immune system cells. In addition to being used to treat TB, the antibiotic rifampicin has also been linked to lower serum calcidiol levels and increased bone turnover. There has been a great enthusiasm to study a link between vitamin D deficiency and the development of TB. Vitamin D deficiency has been established in patients with active TB by several studies. Ninety percent of the seemingly healthy subjects in Delhi were found to be either vitamin D deficient or insufficient, based on serum 25(OH)D cut-off levels of 20 ng/ml and 32 ng/ml, respectively, according to studies conducted across the nation. The activation of TB has long been linked to vitamin D deficiency [25(OH)D] (G and Gupta, 2014). Compared to healthy controls, patients with TB have lower serum levels of vitamin D. There is inconsistent evidence that longer TB treatment lowers serum vitamin D levels as well. According to a number of studies, vitamin D functions as a cofactor to induce antimycobacterial activity, which makes it a powerful immunomodulator of innate immune responses. Patients with TB were advised to receive treatment and rest at sanatoriums with plenty of sunshine before the development of effective antitubercular therapy. The use of cod liver oil for TB treatment was first recommended in 1770 and persisted until the 19th century. In the late 1940s, calciferol was used to treat TB, with the rationale being that it contributed to the calcification of TB lesions. It was thought to be useful in the management of skin TB. After potent anti-tuberculosis medications were developed in the middle of the 1950s, interest in using vitamin D to treat TB decreased. Nonetheless, it served as the foundation for later research that connected Asian Indians’ vulnerability to PTB to a vitamin D deficiency. TB patients had significantly lower levels of 25(OH)D when compared to healthy controls in their comparison of untreated TB patients’ levels. shown in a related study that patients’ radiographic disease was less severe when their 25(OH)D was higher (Hornsby et al., 2018).

### Effect of seasonal variation in vitamin D levels

In contrast to other respiratory diseases, a significant seasonal variation in TB incidence, peaking in the summer and subsequently declining in the spring and winter. They discovered that seasonal variations had an impact on vitamin D levels, which is why TB has a paradoxical reversed seasonality. Sunlight is the main source of vitamin D, and plasma concentrations of the nutrient are known to exhibit dramatic seasonal variation, peaking after summer and falling in the spring (Wingfield et al., 2014). The occurrence of TB has been reported to be related to the seasonal variation in vitamin D levels is prominent in some countries. Since it accounts for both the total amount of vitamin D derived from food and sun exposure, as well as the conversion of vitamin D from adipose stores in the liver, the serum 25(OH)D level is the most accurate measure of overall vitamin D status. There is intense research being done on the connection between vitamin D and tuberculosis. Numerous studies have shown that patients with active tuberculosis also lack vitamin D. 90 percent of the seemingly healthy participants in one study in Delhi were categorized as either vitamin D deficient or insufficient based on serum 25(OH)D cut-off values of 20 ng/ml and 32 ng/ml, respectively. Deficiency of vitamin D [25(OH)D] has long been implicated in the activation of TB. Serum levels of vitamin D in TB patients are lower than in healthy controls. Ironically, extended TB treatment also lowers serum vitamin D levels. According to a number of studies, vitamin D functions as a cofactor to induce antimycobacterial activity, which makes it a powerful immunomodulator of innate immune responses. Patients with TB were advised to receive treatment and recuperation in a sanatorium with plenty of sunlight prior to the development of effective anti-tubercular therapy. As per research conducted by Rathored et al, 2023 serum vitamin D levels, food consumption, and sun exposure were found to be significantly correlated in MDR-TB, DS-PTB, and healthy control individuals. Serum levels may be influenced more by dietary consumption than by sun exposure (Rathored et al., 2024).

### Vitamin D as an adjunct therapy in tuberculosis

The role of vitamin D has been recognized to be crucial in TB. In the 1800s, vitamin D therapy, in the form of cod-liver oil and sunlight exposure, was prevalent as treatment for TB patients. This treatment was victorious in treating patients with lupus vulgaris. Though numerous side effects transpire, such as renal failure and liquefaction of previously silent pulmonary lesions. These are partially due to enhanced release of tissue-destructive macrophage products, such as TNF, which incites necrosis in mycobacterial lesions (Papagni et al., 2022).

Twenty-four newly diagnosed tuberculous children were included in the study, and they were randomly assigned to one of two groups based on the treatment that was given. Patients in group A received only ATT, while patients in group B also received vitamin D **Table 3**. They discovered that group B patients had a more pronounced clinical improvement than group A patients, and this was also evident in the sonographic and X-ray results. In a double-blind, randomized trial, the group receiving 10,000 IU of vitamin D daily for six weeks had significantly higher rates of sputum conversion to culture-negative than the placebo group. Furthermore, compared to the placebo group, a higher number of subjects showed radiological improvement in the vitamin D group. Recently, vitamin D supplementation has been studied as an adjuvant therapy for active TB in two randomized controlled trials. Due to an insufficient dose of vitamin D, clinical outcome did not improve among patients with TB, and showed no overall effect on mortality in patients with TB. However, a recent study shows that when four doses of 2.5 mg vitamin D_3_ were administered to TB patients during the intensive phase, serum 25(OH)D concentrations were increased, which did not significantly affect time to sputum culture conversion; it significantly accelerated sputum culture conversion of patients having *tt* genotype of the *TaqI* VDR polymorphism. According to recent research, patients with sputum bacteriology conversion had a worse prognosis than those with consistently positive sputum for multidrug-resistant tuberculosis (MDR-TB). Two months into a treatment regimen, sputum smear conversions may be a useful indicator of an MDR-TB patient’s prognosis.

### Vitamin D receptor gene

The gene of VDR is located in the long arm of chromosome 12. Vitamin D functions by means of the nuclear hormone receptor known as VDR. Thus, polymorphisms in the VDR gene were investigated as possible candidates for risk markers for a range of clinical outcomes because they may affect VDR activity and ensuing downstream effects mediated by vitamin D. In 2005, a meta-analysis was conducted to assess the association of PTB with VDR *FokI* and *TaqI* polymorphisms. The nucleotide T to C substitution (ATT→ATC) leads to a silent transition at codon 352 in exon 9 is detected by the restriction enzyme *Taq*I. Variants *ApaI* and *BsmI* are both located in introns between exons VIII and IX, and are known to produce splicing errors; therefore, they are unlikely to have functional consequences.

Since both *TaqI* polymorphisms code for isoleucine at amino acid 352, it is unlikely that they will affect VDR function. The longer f allele may be less active in a transfected cell system, and the structural difference resulting from the two alleles may affect the function of the VDR protein. The *Fok*I variant is the functional candidate that is most likely to function. Researchers have found no evidence of allele-specific variations in VDR mRNA, not even a correlation between lower levels of VDR mRNA and the *“t”* allele. Other research, however, suggests that the *TaqI* might have an impact on the balance between Th1 and Th2. Th1-type immune responses are typically produced by homozygotes for *“tt”*, whereas homozygotes produce Th2-type responses for *“TT”.* It has been discovered that people who are homozygous for *“tt”* are resistant to PTB, suggesting that the Th1-type immune response protects against TB. By modifying target gene expression, the VDR is known to mediate the pleiotropic biological actions of 1,25(OH)2 D3. It is reported that both the transcriptional and posttranslational levels regulate this ligand-activated cellular transcription factor.

### Gene organization

The VDR has a gene that consists of a complex intron/exon structure and has been mapped to the long arm of the human chromosome 12 (12cen-q12). Szpirer mapped it to 12q13-14, but 12cen–q12 might be a more centromeric location. As seen in **Figure 2**, it spans roughly 75 kilobases of genomic DNA and has 15 exons. The 5’ untranslated region of the hVDR mRNA is encoded by exons IA through IF, which are known to undergo alternative splicing. The translation start site, which is the DNA binding domain and has two zinc finger motifs (one in each domain), is encoded by exons II and III. The strong heterodimerization domains and the overlapping ligand-binding region are encoded by exons VI–IX. The entire 3’ UTR region is also contained in Exon IX (Martineau et al., 2011), **Figure 2**.

### Gene variants

In numerous ethnic groups, the VDR gene has been identified as extremely polymorphic in nature. 22 mutations have been reported to result in loss of function in the VDR gene. The majority of ligand-binding domains and DNA alterations result in single-nucleotide substitutions. The SNPs examined in this study include: *FokI, BsmI*, and *TaqI*. They are identified by the matching restriction enzymes that identify the variation in them. When alleles are represented by a capital letter, the site is absent, and when it is represented by a lower case, it is present. The nucleotide sequence that is detected by the various restriction enzymes is indicated in **Table 4**.

**Table 4:** Comparison of VDR genotype frequencies in pulmonary tuberculosis in various studies.

References		Countries	Sample size	VDR genotype studied	p-value	Result genotypic frequencies
Shah S et al. (2023) (Shah et al., 2024)		India	TB (n=42) Healthy controls (n=17)	FokI Protective genotype: FF TaqI No statistically significant ApaI Protective genotype: aa BsmI Protective genotype: BB	<0.05	No statistically significant finding regarding TB susceptibility and Taq1 polymorphisms. The development of a TB infection was prevented by VDR polymorphisms in Fok1 and Bsm1, whereas TB susceptibility seemed to be significantly correlated with Apa1 variants.
Yadav U et al. (2021) (Yadav et al., 2021)		South Asia	44 articles meta-analysis	***FF Ff ff*** 40% 45% 15% ***TT Tt tt*** 30% 50% 20% ***AA Aa aa*** 55% 40% 5%	<0.05	Variation in VDR genotype frequencies among TB patients across different populations
Kafle S et al. (2020) (Kafle et al., 2021)		Worldwide	26 studies qualitative synthesis 12 studies in meta-analysis	***BB Bb bb*** 25% 50% 25% ***TT Tt tt*** 35% 45% 20%	>0.05	Lack of significant association between VDR genotypes and TB susceptibility observed
Zhang J-W et al. (2017) (Zhang et al., 2017)		Chinese Han	TB (n=100) Healthy controls (n=100)	***TT Tt tt*** 45% 40% 15% ***AA Aa aa*** 60% 35% 5%	<0.01	Significantly lower frequency of certain VDR genotypes in TB cases reported
Rathored J et al. (2012) (Rathored et al., 2012)		North Indian	MDR-TB (n=354) PTB Cat I (n=338) Healthy controls (n=205)	***TT Tt tt*** 42% 40% 18% 42% 42% 16% 47% 39% 14% ***FF Ff ff*** 41% 51% 8% 52% 34% 14% 58% 39% 3% ***BB Bb bb*** 34% 40% 26% 21% 61% 18% 25% 53% 22%	0.5 <0.001 <0.001	Genotypic frequencies were not significantly different between patients and healthy controls but allelic frequencies were found significant among the three different groups. Allelic and genotypic frequencies were found significantly different between patients and healthy controls Genotypic frequencies were found significantly different between patients and healthy controls
Martineau et al. (2011) (Martineau et al., 2011)		British (London, UK)	PTB Cat I (vitamin D) (n=62) PTB Cat I placebo (n=64)	***TT Tt tt*** 48% 44% 8% 45% 44% 11% ***FF Ff ff*** 47% 40% 13% 42% 38% 20%	0.14 0.85	Allelic and genotypic frequencies were not significantly different in vitamin D and placebo group
Babb et al. (2007) (Babb et al., 2007)	South African admixed		PTB Cat I (n=249) Healthy controls (n=352)	***TT Tt tt*** 55% 38% 8% 54% 40% 6% ***FF Ff ff*** 53% 42% 5% 58% 37% 6%	0.75 0.44	Allelic and genotypic frequencies were not significantly different between patients and healthy controls
Roth et al. (2004) (Roth et al., 2004)	Peruvian		PTB Cat I (n=103) Healthy controls (n=206)	***TT Tt tt*** 90% 10% 0% 83% 17% 0% ***FF Ff ff*** 9% 32% 59% 7% 36% 57%	NS NS	Allelic and genotypic frequencies were not significantly different between patients and healthy controls in VDR *TaqI, Fok*I and *Bsm*I.
Wilkinson et al. (2000) (Wilkinson et al., 2000)	Gujarati Asians (London, UK)		PTB Cat I (n=91) Healthy controls (n=116)	***TT Tt tt*** 43% 51% 6% 39% 50% 11% ***FF Ff ff*** 57% 34% 9% 64% 34% 2%	0.49 0.13	Allelic and genotypic frequencies were not significantly different between patients and healthy controls in VDR *TaqI* and *Fok*I

PTB Cat I, pulmonary tuberculosis Category I; MDR, multi-drug resistant.

BB. TT, FF is homozygous wild type allele, Bb. Tt, Ff is heterozygous allele and bb, tt and ff is homozygous mutant type allele.

In exon two, at the first of two putative translation initiation sites, *FokI* indicates a C-T (ACG – ATG) transition. A person carrying the C allele (designated F) lacks the three NH2-terminal amino acids of the full-length VDR protein and starts translation at the second ATG site. On the other hand, a person carrying the *T* allele, which is denoted as *f*, starts translation at the first ATG site and produces a complete VDR protein. A T-C change, where the *T* allele is designated *B* and the *C* allele is designated b, is caused by *BsmI* found in intron 8. The T-G change caused by the *ApaI* SNP, which is located in intron 8, designates the *G* allele as a and the T allele as A (Elhoseiny et al., 2016).

### Association of VDR polymorphism in tuberculosis

Several studies have been reported on SNPs of the VDR gene (**Table 4**), which is thought to confer genetic differences in vitamin D physiology, susceptibility to active TB development, and TB treatment response (**Table 3A, B, C**). There is strong evidence to suggest that VDR polymorphisms may be related to the development of TB. These polymorphisms might, however, have distinct functions in various populations. Vitamin D works by attaching itself to its receptor. Hence, VDR polymorphisms have been proposed as possible indicators of host susceptibility to TB since they control the receptor’s activity. Allelic variants of the VDR have been found to be associated with differential susceptibility to TB and other infectious diseases. Conducted a study in the Gambian population that included 400 controls and 400 smear-positive TB cases. They observed that the *tt* genotype was significantly underrepresented in control subjects (odds ratio 0.53; 95% CI, 0.31-0.88).

In South Indian patients who were male subjects of Indo-Dravidian descent, the polymorphisms of the VDR gene, specifically *BsmI, ApaI, FokI*, and *TaqI*, were found to be associated with either susceptibility or resistance to PTB. This shows that a male’s susceptibility to PTB may be related to the *Bb* and *FF* genotypes. In contrast, no correlation was found between the *BB* and *AA* genotypes and PTB resistance in male subjects and PTB susceptibility or resistance in female patients. However, it has been demonstrated that in Gujarati Indians residing in London, the variant *ff* genotype of the *FokI* polymorphism of the VDR gene and 25(OH)D deficiency are strongly associated with PTB. In a study of PTB patients in the Gambian population (West Africa), the *tt* genotype of the *TaqI* polymorphism of the VDR gene was found less frequently in cases of PTB, indicating that this genotype may be associated with resistance to PTB, while the *ApaI* polymorphism showed no association. This implies that gene-environment interaction may be the cause of the variant VDR genotypes correlation with various ethnic populations. Numerous studies have hypothesized that vitamin D status may influence the relationship between VDR polymorphisms and disease susceptibility. Twenty-three studies addressing the most extensively studied VDR polymorphisms (*FokI, TaqI, ApaI*, and *BsmI*) are included in their meta-analysis of the relationships between VDR polymorphisms and TB susceptibility. They found Significant associations for *FokI* and *BsmI* polymorphisms among Asians, but none of the polymorphisms were significantly related to TB among Africans or South Americans. However, male patients showed an increased frequency of the heterozygous genotype *Bb* of *BsmI* and *FF* genotype of *FokI* polymorphisms of VDR genes. This suggests that *Bb* and *FF* genotypes may be associated with susceptibility to PTB, and *BB* and *AA* genotypes are associated with resistance to PTB in male subjects. Whereas no association with *BsmI, ApaI*, or *FokI* was found to correlate significantly with the susceptibility or resistance to PTB in female subjects.

The *tt* genotype was linked to higher *in vitro* reporter gene mRNA levels. A study carried out among West London’s Gujarati Asian community did not observe this association, but concluded that a combination of genotypes *TT/Tt* and vitamin D deficiency was associated with TB susceptibility. The VDR *Fok* ‘*ff*’ polymorphism was associated with susceptibility to PTB in a Chinese Han population. In India, it was observed that there was no association between the *Taq*I genotype and TB susceptibility or resistance; however, the homozygous ‘*tt*’ genotype was overrepresented in females. In 1999, tested association with leprosy in a case-control population from Mali and in families from South India. There was no significant difference between the leprosy cases and the controls in the analysis of genotypes. However, a significant increase (p-value<0.001) in the *tt* genotype was observed amongst tuberculoid leprosy cases when compared to healthy controls from Calcutta. The genotype *TT* was over-represented in the lepromatous leprosy cases (p-value = 0.03), possibly indicating an association between *tt* and a Th1 type of response, and *TT* and a Th2 type of response **Table 3**.

## Discussion

As vitamin D affects T-cells, antimicrobial peptides, and macrophages, it plays a significant role in immunological modulation in tuberculosis (TB). The examination of vitamin D levels and VDR polymorphisms in tuberculosis is supported by this. But comprehending its clinical importance, particularly in multidrug-resistant tuberculosis (MDR-TB), necessitates a rigorous distinction between the disease’s specific data and theoretical causes. Research mostly on drug-susceptible individuals has shown that low blood vitamin D levels are associated with an increased risk of active pulmonary tuberculosis (TB) (Bavi et al., 2024). Although impact sizes vary according to region, ethnicity, nutritional status, and research design, these correlations consistently show up in many circumstances. These connections do not, however, suggest causality because vitamin D deficiency may not directly cause tuberculosis (TB) but rather reflect other variables including disease load, hunger, or socioeconomic problems. There is limited information on vitamin D status and VDR polymorphisms in MDR-TB, and it frequently comes from observational studies that are either underpowered or combined with TB patients that are amenable to drugs. There have been reports of lower vitamin D levels in MDR-TB patients; however, it is still unknown if this deficit is a result of treatment-related metabolic changes, protracted illness, or drug resistance (Amrein et al., 2020).

There is considerable variation in the relationships between common VDR polymorphisms and tuberculosis (TB) outcomes. In some genotypic subgroups, especially the TaqI tt genotype, some signals show quicker sputum or culture conversion with vitamin D treatment; however, these results are not consistently detected across investigations and are frequently missing without genotype stratification. No general impact of vitamin D supplementation on culture conversion, mortality, or end-of-treatment outcomes has been identified in a number of randomized controlled studies, indicating that any possible benefit is probably context-dependent (Cao et al., 2015).

**Figure 5:**
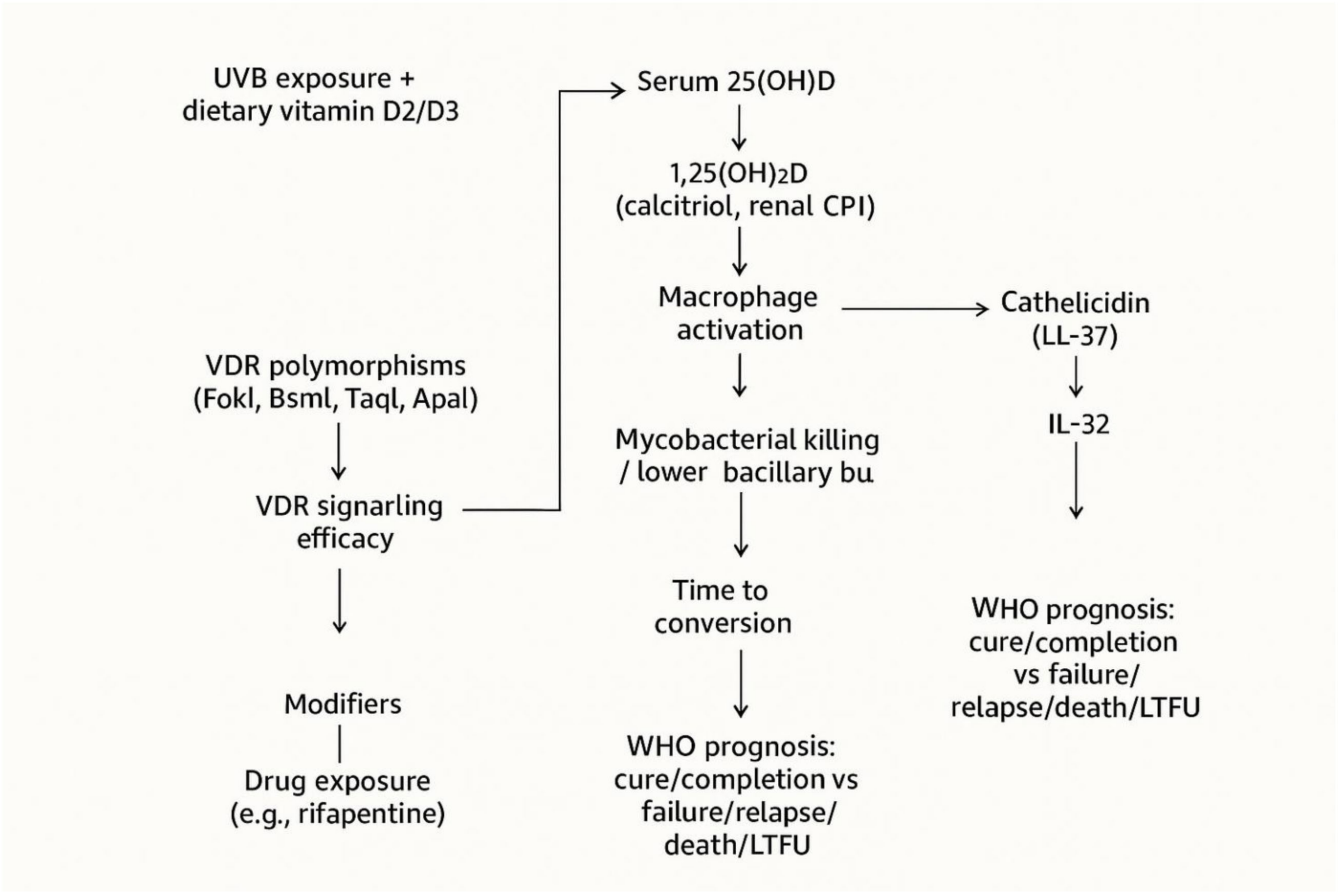
Host vitamin D signaling and VDR polymorphisms shaping immune response and MDR-TB outcomes. Inputs (UVB, diet) raise 25(OH)D and calcitriol, while VDR polymorphisms modulate signaling. Together they drive immune effectors that reduce bacillary burden, accelerating culture conversion and improving WHO-defined outcomes. Clinical modifiers (drug exposure, cavities, comorbidities) attenuate clearance. UVB, ultraviolet B; 25(OH)D, 25-hydroxyvitamin D; 1,25(OH)2D, 1,25-dihydroxyvitamin D (calcitriol); CYP27B1, 1-α-hydroxylase; VDR, vitamin D receptor; IL, interleukin; IFN-γ, interferon-gamma; T2DM, type 2 diabetes mellitus; LTFU, loss to follow-up; WHO, World Health Organization.

Mechanistic distinction for MDR-TB: VDR-mediated induction of antimicrobial peptides and macrophage antimicrobial functions is broadly relevant to TB, but may be especially consequential in MDR-TB where reduced drug bactericidal activity heightens reliance on host-mediated killing; PK–PD and disease-burden determinants (e.g., rifapentine exposure and large cavities) further shape time to conversion, implying that host-directed effects will be most evident when drug exposure is adequate yet clearance remains host-limited (Mehta et al., 2022) (**Figure 5**).

This review interprets heterogeneous findings through a unifying pathway: vitamin D status and VDR variants shape macrophage-mediated effector responses, while programmatic modifiers alter clearance, together influencing culture conversion and WHO-defined outcomes (**Figure 3**). Presenting this schematic upfront in the Discussion supports readers in connecting mechanistic plausibility to the standardized outcome definitions used across included studies.

Common VDR polymorphisms (FokI, BsmI, TaqI, ApaI) may influence VDR activity and downstream immune effects, and associations with TB susceptibility or treatment response vary by ancestry, study design, and baseline vitamin D status, underscoring potential effect modification by genotype and environment (Kafentzi et al., 2025). Taken together, the literature indicates a complex interplay of genetics, vitamin D metabolism, and immune response in TB, with more consistent signals for susceptibility than for MDR-TB treatment outcomes, and with genotype–response interactions that merit targeted evaluation in adequately powered, stratified cohorts and trials (**Table 5A**) (Bavi et al., 2024).

**Table 5A:** Restriction enzyme recognition based on SNPs in VDR gene.

Restriction enzyme	Allele	Sequence	Recognized by enzyme
***FokI***	F	5′ GGACG 3′ 3′ CCTGC (N)_13_ 5′	✗
	f	5′ GGATG (N)_9_ ↓3′ 3′ CCTAC (N)_13_ 5′	✓
***BsmI***	B	5′ GAATACNN 3′ 3′ CTTATGNN 5′	✗
	b	5′ GAATGCNJN 3′ 3′ CTTAC↓GNN 5′	✓
***TaqI***	T	5′ TCAA 3′ 3′ AGTT 5′	✗
	t	5′ T↓CGA 3′ 3′ AGC↑T 5′	✓

SNP = single nucleotide polymorphism, VDR = vitamin D receptor.

✗=not recognized, ✓=recognized.

Fok 1, Bsm 1 and Taq 1 are the restriction sites of VDR polymorphisms.

**Table 5B:** Summary of key studies on VDR polymorphisms relevant to MDR-TB.

Study (year)	Population	MDR-TB sample	Variant(s)	Outcome(s)	Key finding
Rathored et al., 2012 (Rathored et al., 2012)	North India clinical cohorts	354	TaqI, FokI, BsmI	Genotype/allele distribution; clinical outcomes	Genotypic frequencies not significantly different versus controls; allelic differences across MDR-TB, DS-TB, and controls reported; supports investigating allele-level effects in MDR-TB populations .
Rathored et al., 2024 (Rathored et al., 2024)	North India programmatic (Cat I and MDR-TB)	Subset MDR	Not genotype-focused; vitamin D status	Time to smear/culture conversion; prognosis	MDR-TB showed slower conversion than DS-TB; two-month smear conversion associated with prognosis; contextualizes where host factors could modify response in MDR-TB .
Martineau et al., 2011 (Martineau et al., 2011)	UK RCT, DS-TB	—	TaqI (effect modification)	Time to culture conversion	No overall benefit of adjunct vitamin D; faster conversion signal in TaqI tt subgroup suggests genotype-dependent effects relevant to future MDR-TB stratification studies .
Roth et al., 2004 (Roth et al., 2004)	Peru cohort, DS-TB	—	FokI, TaqI, BsmI	Smear/culture conversion	VDR genotypes associated with conversion speed; informs mechanistic plausibility for genotype-stratified designs in MDR-TB .
Babb 2007 (Babb et al., 2007)	South Africa cohort, DS-TB	—	Multiple VDR SNPs	Fast (<2 mo) vs slow responders	Genetic variation related to conversion phenotype; supports exploring responder strata in MDR-TB .

Translational relevance: Vitamin D supplementation is low-cost and scalable but shows mixed overall efficacy; a pragmatic path in high-burden programs is deficiency testing with targeted supplementation and prospective evaluation of genotype-stratified effects rather than universal adjunct use, while selective VDR genotyping (FokI/TaqI/BsmI/ApaI) is feasible where molecular capacity exists and can be embedded within MDR/RR-TB care (e.g., BPaL/BPaLM) with routine safety monitoring, adherence support, and standardized WHO outcomes before wider implementation (Singh et al., 2025) (**Table 5B**).

Given these patterns, future work should standardize outcome definitions (e.g., culture conversion and WHO prognosis categories), prespecify confounders (HIV, diabetes, nutrition), and test genotype-stratified adjunctive strategies aligned with contemporary MDR/RR-TB care models to clarify clinical utility.

### Contextualizing VDR polymorphisms within contemporary TB host genetics

Recent developments in the genetics of tuberculosis (TB) hosts suggest that a polygenic and pathway-level immunological architecture, rather than individual candidate genes acting alone, shapes disease susceptibility, progression, and treatment response (Dashian et al., 2025). Genes such as HLA-DRB1, SLC11A1 (NRAMP1), TLR2, TLR4, IFNG, IL12B, and TNF are among the loci involved in innate immune signaling, antigen presentation, macrophage activation, and cytokine regulation that have been repeatedly identified by genome-wide association studies (GWAS) and large candidate-gene consortia. Vitamin D receptor (VDR) polymorphisms are best understood in this larger context as immune response modifiers rather than as the main factors influencing TB or MDR-TB outcomes (Jafari et al., 2016).

By controlling autophagy, cathelicidin (LL-37) production, and macrophage antimicrobial activity downstream of Toll-like receptor activation, VDR signaling interacts mechanistically with recognized TB host-defense mechanisms. Therefore, under situations of vitamin D sufficiency or insufficiency, VDR variations like FokI, TaqI, BsmI, and ApaI may affect the amount and effectiveness of host-mediated mycobacterial clearance. The impact sizes of specific VDR SNPs, however, appear to be small and highly context-dependent, influenced by environmental exposures, comorbidities, nutritional status, and ancestry, according to recent genetic findings (Meca et al., 2021).

Significantly, the strongest genetic correlations in tuberculosis have been found for disease susceptibility rather than treatment response; this distinction is particularly important for MDR-TB. While host genetics, including VDR variations, probably impact treatment kinetics (e.g., culture conversion) and immunological recovery rather than directly influencing resistance mechanisms, drug resistance is predominantly a pathogen-driven phenomena. Therefore, current host-genetic models, which anticipate gradual, modifying effects rather than clinically decisive genetic markers, are consistent with the sparse and inconsistent MDR-TB-specific findings on VDR polymorphisms (Aravindan, 2019).

Faster sputum or culture conversion in TaqI tt carriers are examples of genotype-specific reactions to vitamin D treatment that emphasize gene-environment interactions in a multifactorial immunological milieu rather than general effects. These findings support contemporary viewpoints that pharmacokinetics, disease load, and immunological phenotype in genetically varied populations must be taken into account for host-directed medicines to be successful (Zhang et al., 2019). VDR polymorphisms are thought to be physiologically significant yet non-deterministic variables that affect immune responses. In order to further understand VDR variations’ roles in treatment success, future MDR-TB research should evaluate them in combination with other immune-regulatory genes using polygenic, pathway-based techniques and standardized clinical outcomes (Sutaria et al., 2014).

### Evidence-supported pathways

Vitamin D deficiency is linked to a higher risk of active pulmonary tuberculosis, supported by various studies, including observational studies and randomized controlled trials. Vitamin D signaling enhances macrophage antibacterial activity through cathelicidin induction and Th1/Th2 modulation. Genotype-specific analyses reveal that certain vitamin D receptor (VDR) variants, notably TaqI tt genotype, correlate with quicker sputum or culture conversion post-supplementation, though this effect varies across different genotypes and populations. Overall, these results indicate a significant role for vitamin D–VDR pathways in the immune response to Mycobacterium tuberculosis.

### Inferential pathways

However, the precise application of these pathways to multidrug-resistant tuberculosis (MDR-TB) is still mostly speculative. Strong MDR-TB-specific clinical investigations have not yet explicitly confirmed the theory that host-directed immune mechanisms controlled by vitamin D and VDR polymorphisms may become more significant in MDR-TB due to the decreased bactericidal effectiveness of second-line medications. Similar to this, inferential relationships between VDR polymorphisms, pharmacokinetic–pharmacodynamic parameters, cavity load, and delayed bacterial clearance in MDR-TB are mostly drawn from mixed cohorts or drug-susceptible TB. Therefore, rather than being supported by solid clinical data, these pathways should be seen as mechanistic theories.

### Integrated interpretation

When considered collectively, the available data clearly indicates that vitamin D level and VDR polymorphisms play a part in immunological regulation and TB susceptibility, although it is yet unclear how these factors affect the prognosis and response to MDR-TB treatment. To prevent overinterpretation, it is crucial to distinguish clearly between theorized processes and evidence-based relationships. To confirm if these proposed routes result in clinically useful improvements, more sufficiently powered, genotype-stratified MDR-TB trials utilizing standardized WHO-defined outcomes are needed.

## Conclusion

This review adds value beyond earlier syntheses by separating evidence strength across domains—consistent signals for TB susceptibility versus limited, heterogeneous evidence for MDR-TB treatment response and prognosis while interpreting findings within WHO-aligned outcomes to improve comparability across studies. The main message is that vitamin D status and common VDR variants (FokI, TaqI, BsmI, ApaI) plausibly shape host immunity and may influence culture conversion and outcomes in specific contexts, but MDR-TB-specific effects remain uncertain and require adequately powered, genotype-stratified studies using standardized culture conversion and prognosis definitions. In practice, a pragmatic approach is deficiency testing with targeted supplementation and piloting selective VDR genotyping where capacity exists, embedded in MDR/RR-TB care pathways with adherence support and WHO-harmonized endpoints to judge added value before routine adoption. Future work should jointly model host factors (vitamin D, VDR genotype), pharmacokinetics/pharmacodynamics, and disease burden to identify when host-directed strategies meaningfully accelerate clearance and improve end-of-treatment outcomes.

## Data Availability

The original contributions presented in the study are included in the article/supplementary material. Further inquiries can be directed to the corresponding author.

## References

[B1] Amos W. Driscoll E. Hoffman J. I. (2011). Candidate genes versus genome-wide associations: which are better for detecting genetic susceptibility to infectious disease? Proc. Biol. Sci. 278, 1183–1188. doi: 10.1098/rspb.2010.1920, PMID: 20926441 PMC3049081

[B2] Amrein K. Scherkl M. Hoffmann M. Neuwersch-Sommeregger S. Köstenberger M. Tmava Berisha A. . (2020). Vitamin D deficiency 2.0: an update on the current status worldwide. Eur. J. Clin. Nutr. 74, 1498–1513. doi: 10.1038/s41430-020-0558-y, PMID: 31959942 PMC7091696

[B3] Aparna P. Muthathal S. Nongkynrih B. Gupta S. K. (2018). Vitamin D deficiency in India. J. Family Med. Prim Care 7, 324–330. doi: 10.4103/jfmpc.jfmpc_78_18, PMID: 30090772 PMC6060930

[B4] Aravindan P. (2019). Host genetics and tuberculosis: Theory of genetic polymorphism and tuberculosis. Lung India 36, 244–252. doi: 10.4103/lungindia.lungindia_146_15, PMID: 31031349 PMC6503728

[B5] . Immigrants and tuberculosis in Hong Kong (Hong Kong medical Journal: HKMJ). Available online at: https://www.hkmj.org/abstracts/v21n4/318.htm (Accessed August 21, 2015). 10.12809/hkmj144492 26183454

[B6] Babb C. Keet E. H. van Helden P. D. Hoal E. G. (2007). SP110 polymorphisms are not associated with pulmonary tuberculosis in a South African population. Hum. Genet. 121, 521–522. doi: 10.1007/s00439-007-0335-1, PMID: 17287948

[B7] Bavi H. Hosseini S. A. Ekrami A. Alavi S. M. Malehi A. S. (2024). Effect of vitamin D supplementation on the treatment of pulmonary tuberculosis patients in different polymorphisms of the vitamin D receptor. Adv. BioMed. Res. 13, 102. doi: 10.4103/abr.abr_76_24, PMID: 39717253 PMC11665168

[B8] Bergwitz C. Jüppner H. (2010). Regulation of phosphate homeostasis by PTH, vitamin D, and FGF23. Annu. Rev. Med. 61, 91–104. doi: 10.1146/annurev.med.051308.111339, PMID: 20059333 PMC4777331

[B9] Bikle D. D. (2000). “ Vitamin D: production, metabolism, and mechanism of action,” in Endotext. Eds. Feingold K. R. Adler R. A. Ahmed S. F. Anawalt B. Blackman M. R. Chrousos G. ( MDText.com, Inc, South Dartmouth (MA). 25905172

[B10] Bikle D. D. (2014). Vitamin D metabolism, mechanism of action, and clinical applications. Chem. Biol. 21, 319–329. doi: 10.1016/j.chembiol.2013.12.016, PMID: 24529992 PMC3968073

[B11] Bishop E L. Ismailova A. Dimeloe S. Hewison M. White J. H. (2021). Vitamin D and immune regulation: antibacterial, antiviral, anti-inflammatory. JBMR Plus 5, e10405. doi: 10.1002/jbm4.10405, PMID: 32904944 PMC7461279

[B12] Cao Y. Wang X. Cao Z. Cheng X. (2015). Association of Vitamin D receptor gene TaqI polymorphisms with tuberculosis susceptibility: a meta-analysis. Int. J. Clin. Exp. Med. 8, 10187–10203., PMID: 26309718 PMC4538126

[B13] Chai Q. Lu Z. Liu C. H. (2019). Host defense mechanisms against Mycobacterium tuberculosis. Cell Mol. Life Sci. 77, 1859–1878. doi: 10.1007/s00018-019-03353-5, PMID: 31720742 PMC11104961

[B14] Chen B. Cole J. W. Grond-Ginsbach C. (2017). Departure from hardy weinberg equilibrium and genotyping error. Front. Genet. 8, 167. doi: 10.3389/fgene.2017.00167, PMID: 29163635 PMC5671567

[B15] Clarke G. M. Anderson C. A. Pettersson F. H. Cardon L. R. Morris A. P. Zondervan K. T. (2011). Basic statistical analysis in genetic case-control studies. Nat. Protoc. 6, 121–133. doi: 10.1038/nprot.2010.182, PMID: 21293453 PMC3154648

[B16] Conradie F. Diacon A. H. Ngubane N. Howell P. Everitt D. Crook A. M. . (2020). Treatment of highly drug-resistant pulmonary tuberculosis. N Engl. J. Med. 382, 893–902. doi: 10.1056/NEJMoa1901814, PMID: 32130813 PMC6955640

[B17] Dashian M. A. Shipulin G. A. Deviatkin A. A. (2025). Genetic susceptibility to tuberculosis and the utility of polygenic scores in population stratification. Int. J. Mol. Sci. 26, 9544. doi: 10.3390/ijms26199544, PMID: 41096810 PMC12524525

[B18] Dominguez L. J. Farruggia M. Veronese N. Barbagallo M. (2021). Vitamin D sources, metabolism, and deficiency: available compounds and guidelines for its treatment. Metabolites 11, 255. doi: 10.3390/metabo11040255, PMID: 33924215 PMC8074587

[B19] Duan T. Du Y. Xing C. Wang H. Y. Wang R. F. (2022). Toll-like receptor signaling and its role in cell-mediated immunity. Front. Immunol. 13, 812774. doi: 10.3389/fimmu.2022.812774, PMID: 35309296 PMC8927970

[B20] Elhoseiny S. M. Morgan D. S. Rabie A. M. Bishay S. T. (2016). Vitamin D receptor (VDR) gene polymorphisms (FokI, bsmI) and their relation to vitamin D status in pediatrics βeta thalassemia major. Indian J. Hematol. Blood Transfus 32, 228–238. doi: 10.1007/s12288-015-0552-z, PMID: 27065588 PMC4789011

[B21] G R. Gupta A. (2014). Vitamin D deficiency in India: prevalence, causalities and interventions. Nutrients 6, 729–775. doi: 10.3390/nu6020729, PMID: 24566435 PMC3942730

[B22] Ghaseminejad-Raeini A. Ghaderi A. Sharafi A. Nematollahi-Sani B. Moossavi M. Derakhshani A. . (2023). Immunomodulatory actions of vitamin D in various immune-related disorders: a comprehensive review. Front. Immunol. 14, 950465. doi: 10.3389/fimmu.2023.950465, PMID: 37520529 PMC10379649

[B23] Girishbhai Patel D. Baral T. Jacob Kurian S. Malakapogu P. Saravu K. Sekhar Miraj S. (2024). Nutritional status in patients with tuberculosis and diabetes mellitus: A comparative observational study. J. Clin. Tuberc Other Mycobact Dis. 35, 100428. doi: 10.1016/j.jctube.2024.100428, PMID: 38549700 PMC10973140

[B24] Gler M. T. Guilatco R. Caoili J. C. Ershova J. Cegielski P. Johnson J. L. (2013). Weight gain and response to treatment for multidrug-resistant tuberculosis. Am. J. Trop. Med. Hyg 89, 943–949. doi: 10.4269/ajtmh.13-0011, PMID: 24019430 PMC3820341

[B25] Griffith D. E. Adjemian J. Brown-Elliott B. A. Philley J. V. Prevots D. R. Gaston C. . (2015). Semiquantitative Culture Analysis during Therapy for Mycobacterium avium Complex Lung Disease. Am. J. Respir. Crit. Care Med. 192, 754–760. doi: 10.1164/rccm.201503-0444OC, PMID: 26068042 PMC4595680

[B26] Gulumbe B. H. Abdulrahim A. Ahmad S. K. Lawan K. A. Danlami M. B. (2025). WHO report signals tuberculosis resurgence: Addressing systemic failures and revamping control strategies. Decoding Infection Transm. 3, 100044. doi: 10.1016/j.dcit.2025.100044

[B27] Harinarayan C. V. Holick M. F. Prasad U. V. Vani P. S. Himabindu G. (2013). Vitamin D status and sun exposure in India. Dermatoendocrinol 5, 130–141. doi: 10.4161/derm.23873, PMID: 24494046 PMC3897581

[B28] Hayeck T. J. Li Y. Mosbruger T. L. Bradfield J. P. Gleason A. G. Damianos G. . (2024). The impact of patterns in linkage disequilibrium and sequencing quality on the imprint of balancing selection. Genome Biol. Evol. 16, evae009. doi: 10.1093/gbe/evae009, PMID: 38302106 PMC10853003

[B29] Hibma J. E. Radtke K. K. Dorman S. E. Jindani A. Dooley K. E. Weiner M. . (2020). Rifapentine population pharmacokinetics and dosing recommendations for latent tuberculosis infection. Am. J. Respir. Crit. Care Med. 202, 866–877. doi: 10.1164/rccm.201912-2489OC, PMID: 32412342 PMC7491398

[B30] Hornsby E. Pfeffer P. E. Laranjo N. Cruikshank W. Tuzova M. Litonjua A. A. . (2018). Vitamin D supplementation during pregnancy: Effect on the neonatal immune system in a randomized controlled trial. J. Allergy Clin. Immunol. 141, 269–278.e1. doi: 10.1016/j.jaci.2017.02.039, PMID: 28552588

[B31] Huang S. J. Wang X. H. Liu Z. D. Cao W. L. Han Y. Ma A. G. . (2016). Vitamin D deficiency and the risk of tuberculosis: a meta-analysis. Drug Des. Devel Ther. 11, 91–102. doi: 10.2147/DDDT.S79870, PMID: 28096657 PMC5207333

[B32] Imperial M. Z. Nahid P. Phillips P. P. J. Davies G. R. Fielding K. Hanna D. . (2018). A patient-level pooled analysis of treatment-shortening regimens for drug-susceptible pulmonary tuberculosis. Nat. Med. 24, 1708–1715. Available online at: https://www.nature.com/articles/s41591-018-0224-2 (Accessed November 24, 2018)., PMID: 30397355 10.1038/s41591-018-0224-2 PMC6685538

[B33] Jafari M. Nasiri M. R. Sanaei R. Anoosheh S. Farnia P. Sepanjnia A. . (2016). The NRAMP1, VDR, TNF-α, ICAM1, TLR2 and TLR4 gene polymorphisms in Iranian patients with pulmonary tuberculosis: A case-control study. Infect. Genet. Evol. 39, 92–98. doi: 10.1016/j.meegid.2016.01.013, PMID: 26774366

[B34] Kafentzi T. Tsounis E. P. Tourkochristou E. Avramopoulou E. Aggeletopoulou I. Geramoutsos G. . (2025). Genetic polymorphisms (ApaI, fokI, bsmI, and taqI) of the vitamin D receptor (VDR) influence the natural history and phenotype of crohn’s disease. Int. J. Mol. Sci. 26, 1848. doi: 10.3390/ijms26051848, PMID: 40076474 PMC11899612

[B35] Kafle S. Basnet A. K. Karki K. Thapa Magar M. Shrestha S. Yadav R. S. (2021). Association of vitamin D deficiency with pulmonary tuberculosis: A systematic review and meta-analysis. Cureus. 13, e17883. doi: 10.7759/cureus.17883, PMID: 34660082 PMC8504877

[B36] Kearns M. D. Tangpricha V. (2014). The role of vitamin D in tuberculosis. J. Clin. Transl. Endocrinol. 1, 167–169. doi: 10.1016/j.jcte.2014.08.002, PMID: 29159097 PMC5684962

[B37] Legesse T. Admenur G. Gebregzabher S. Woldegebriel E. Fantahun B. Tsegay Y. . (2021). Tuberculosis (TB) in the refugee camps in Ethiopia: trends of case notification, profile, and treatment outcomes, 2014 to 2017. BMC Infect. Dis. 21, 139. doi: 10.1186/s12879-021-05828-y, PMID: 33535974 PMC7856765

[B38] Lin H. W. Chen Y. H. (2010). Association analysis under population stratification: A two-stage procedure utilizing population- and family-based analyses. Hum. Hered. 69, 160–170. doi: 10.1159/000267996, PMID: 20029228

[B39] Liu T. Wang L. Ou L. Feng J. Cheng J. Gong Z. (2025). Impact of whole-grain interventions on serum vitamin D levels in individuals at risk of diabetes. Front. Nutr. 12, 1658961. doi: 10.3389/fnut.2025.1658961, PMID: 41211221 PMC12588831

[B40] Lonnie M. Hooker E. Brunstrom J. M. Corfe B. M. Green M. A. Watson A. W. . (2018). Protein for life: review of optimal protein intake, sustainable dietary sources and the effect on appetite in ageing adults. Nutrients 10, 360. doi: 10.3390/nu10030360, PMID: 29547523 PMC5872778

[B41] Lu J. T. Ilyas E. (2022). An overview of ultraviolet-protective clothing. Cureus 14, e27333. doi: 10.7759/cureus.27333, PMID: 36043025 PMC9414157

[B42] Martineau A. R. Timms P. M. Bothamley G. H. Hanifa Y. Islam K. Claxton A. P. . (2011). High-dose vitamin D(3) during intensive-phase antimicrobial treatment of pulmonary tuberculosis: a double-blind randomised controlled trial. Lancet. 377, 242–250. doi: 10.1016/S0140-6736(10)61889-2, PMID: 21215445 PMC4176755

[B43] Mase S. R. Chorba T. (2019). Treatment of drug-resistant tuberculosis. Clin. Chest Med. 40, 775–795. doi: 10.1016/j.ccm.2019.08.002, PMID: 31731984 PMC7000172

[B44] Meca A. D. Ștefănescu S. Bogdan M. Turcu-Știolică A. Nițu F. M. Matei M. . (2021). Crosstalk between vitamin D axis, inflammation and host immunity mechanisms: A prospective study. Exp. Ther. Med. 21, 608. doi: 10.3892/etm.2021.10040, PMID: 33936265 PMC8082620

[B45] Mehta K. Sharma P. Mujawar S. Vyas A. (2022). Role of antimicrobial peptides in treatment and prevention of mycobacterium tuberculosis: A review. Int. J. Pept. Res. Ther. 28, 132. doi: 10.1007/s10989-022-10435-9, PMID: 35891800 PMC9305673

[B46] Menon P. M. Chandrasekaran N. Doss C G. P. (2024). Investigating the impact of first-line anti-tuberculosis drugs encapsulated in a eugenol-based nanoemulsion on human serum albumin. Discov. Med. 36, 739–752. doi: 10.24976/Discov.Med.202436183.70, PMID: 38665023

[B47] Mily A. Rekha R. S. Kamal S. M. M. Arifuzzaman A. S. M. Rahim Z. Khan L. . (2015). Significant effects of oral phenylbutyrate and vitamin D3 adjunctive therapy in pulmonary tuberculosis: A randomized controlled trial. PloS One 10, e0138340. doi: 10.1371/journal.pone.0138340, PMID: 26394045 PMC4578887

[B48] Möller M. Kinnear C. J. Orlova M. Kroon E. E. van Helden P. D. Schurr E. . (2018). Genetic resistance to mycobacterium tuberculosis infection and disease. Front. Immunol. 9, 2219. doi: 10.3389/fimmu.2018.02219, PMID: 30319657 PMC6170664

[B49] Morales F. Montserrat-de la Paz S. Leon M. J. Rivero-Pino F. (2023). Effects of malnutrition on the immune system and infection and the role of nutritional strategies regarding improvements in children’s health status: A literature review. Nutrients 16, 1. doi: 10.3390/nu16010001, PMID: 38201831 PMC10780435

[B50] Nahid P. Horne D. J. Jarlsberg L. G. Reiner A. P. Osmond D. Hopewell P. C. . (2011). Racial differences in tuberculosis infection in United States communities: the coronary artery risk development in young adults study. Clin. Infect. Dis. 53, 291–294. doi: 10.1093/cid/cir378, PMID: 21765079 PMC3137794

[B51] Papagni R. Pellegrino C. Di Gennaro F. Patti G. Ricciardi A. Novara R. . (2022). Impact of vitamin D in prophylaxis and treatment in tuberculosis patients. Int. J. Mol. Sci. 23, 3860. doi: 10.3390/ijms23073860, PMID: 35409219 PMC8999210

[B52] Rathored J. Sharma S. K. Banavaliker J. N. Sreenivas V. Srivastava A. K. (2024). Response to treatment and low serum vitamin D levels in North Indian patients with treatment-naive category I and multi-drug resistant pulmonary tuberculosis. Ann. Med. 56, 2407066. doi: 10.1080/07853890.2024.2407066, PMID: 39311013 PMC11421155

[B53] Rathored J. Sharma S. K. Singh B. Banavaliker J. N. Sreenivas V. Srivastava A. K. . (2012). Risk and outcome of multidrug-resistant tuberculosis: vitamin D receptor polymorphisms and serum 25(OH)D. Int. J. Tuberc Lung Dis. 16, 1522–1528. doi: 10.5588/ijtld.12.0122, PMID: 22990231

[B54] Rathored J. Sharma S. K. Sreenivas V. Srivastava A. K. (2025). Association of vitamin D receptor mRNA expression, vitamin D deficiency and genetic variant in patients with multi-drug resistant pulmonary tuberculosis. BMC Infect. Dis. 25, 1334. doi: 10.1186/s12879-025-11707-7, PMID: 41094671 PMC12522335

[B55] Roque-Borda C. A. Vishwakarma S. K. Ramirez Delgado O. J. de Souza Rodrigues H. L. Primo L. M. D. Campos I. C. . (2025). Peptide-based strategies against mycobacterium tuberculosis covering immunomodulation, vaccines, synergistic therapy, and nanodelivery. Pharmaceuticals 18, 1440. doi: 10.3390/ph18101440, PMID: 41155557 PMC12566800

[B56] Roth D. E. Soto G. Arenas F. Bautista C. T. Ortiz J. Rodriguez R. . (2004). Association between vitamin D receptor gene polymorphisms and response to treatment of pulmonary tuberculosis. J. Infect. Dis. 190, 920–927. doi: 10.1086/423212, PMID: 15295697

[B57] Sekhar Miraj S. Vyas N. Kurian S. J. Baral T. Thomas L. Reddy B. S. . (2022). Vitamin D receptor gene polymorphism and vitamin D supplementation on clinical/ treatment outcome in tuberculosis: current and future perspectives. Expert Rev. Anti Infect. Ther. 20, 1179–1186. doi: 10.1080/14787210.2022.2081546, PMID: 35608034

[B58] Shah S. Priyanka Sharma S. (2024). An updated trial sequential meta-analysis of vitamin D receptor gene polymorphism (Fok1, bsm1, taq1 and apa1) and risk to tuberculosis. Indian J. Clin. Biochem. 39, 60–72. doi: 10.1007/s12291-022-01091-3, PMID: 38223006 PMC10784437

[B59] Singh S. Meena R. K. Maharshi V. Sinha N. Agarwal N. Payra S. . (2025). Vitamin D supplementation trials: Navigating the maze of unpredictable results. Perspect. Clin. Res. 16, 69–74. doi: 10.4103/picr.picr_325_23, PMID: 40322473 PMC12048097

[B60] Supriya R. Gao Y. Gu Y. Baker J. S. (2021). Role of exercise intensity on th1/th2 immune modulations during the COVID-19 pandemic. Front. Immunol. 12, 761382. doi: 10.3389/fimmu.2021.761382, PMID: 35003073 PMC8727446

[B61] Sutaria N. Liu C. T. Chen T. C. (2014). Vitamin D status, receptor gene polymorphisms, and supplementation on tuberculosis: A systematic review of case-control studies and randomized controlled trials. J. Clin. Transl. Endocrinol. 1, 151–160. doi: 10.1016/j.jcte.2014.08.001, PMID: 25599020 PMC4295520

[B62] Theodoropoulos C. Demers C. Petit J. L. Gascon-Barré M. (2003). High sensitivity of rat hepatic vitamin D3–25 hydroxylase CYP27A to 1,25-dihydroxyvitamin D3 administration. Am. J. Physiology-Endocrinology Metab. 284, 1–53 doi: 10.1152/ajpendo.00303.2002, PMID: 12388126

[B63] Tiwari S. Kumar A. Kapoor S. K. (2012). Relationship between sputum smear grading and smear conversion rate and treatment outcome in the patients of pulmonary tuberculosis undergoing dots–a prospective cohort study. Indian J. Tuberc. 59, 135–140., PMID: 23362709

[B64] Torres N. M. C. Rodriguez J. J. Q. Andrade P. S. P. Arriaga M. B. Netto E. M. (2015). (PDF) Factors predictive of the success of tuberculosis treatment: A systematic review with meta-analysis ( Hongkong Medical Journal: ResearchGate). Available online at: https://www.researchgate.net/publication/338205830_Factors_predictive_of_the_success_of_tuberculosis_treatment_A_systematic_review_with_meta-analysis (Accessed August 21, 2015).

[B65] Tourkochristou E. Tsounis E. P. Tzoupis H. Aggeletopoulou I. Tsintoni A. Lourida T. . (2023). The influence of single nucleotide polymorphisms on vitamin D receptor protein levels and function in chronic liver disease. Int. J. Mol. Sci. 24, 11404. doi: 10.3390/ijms241411404, PMID: 37511164 PMC10380285

[B66] Uçar N. Holick M. F. (2025). Illuminating the connection: cutaneous vitamin D3 synthesis and its role in skin cancer prevention. Nutrients 17, 386. doi: 10.3390/nu17030386, PMID: 39940244 PMC11821240

[B67] Usategui-Martín R. De Luis-Román D. A. Fernández-Gómez J. M. Ruiz-Mambrilla M. Pérez-Castrillón J. L. (2022). Vitamin D receptor (VDR) gene polymorphisms modify the response to vitamin D supplementation: A systematic review and meta-analysis. Nutrients 14, 360. doi: 10.3390/nu14020360, PMID: 35057541 PMC8780067

[B68] Varughese M. Heffernan C. Li M. Y. Long R. (2023). Time to diagnosis and treatment of pulmonary tuberculosis in indigenous peoples: a systematic review. BMC Infect. Dis. 23, 131. doi: 10.1186/s12879-023-08098-y, PMID: 36882707 PMC9989566

[B69] Velayutham B. R. V. Nair D. Chandrasekaran V. Raman B. Sekar G. Watson B. . (2014). Profile and Response to Anti-Tuberculosis Treatment among Elderly Tuberculosis Patients Treated under the TB Control Programme in South India. PloS One 9, e88045. doi: 10.1371/journal.pone.0088045, PMID: 24618888 PMC3949670

[B70] Voltan G. Cannito M. Ferrarese M. Ceccato F. Camozzi V. (2023). Vitamin D: an overview of gene regulation, ranging from metabolism to genomic effects. Genes 14, 1691. doi: 10.3390/genes14091691, PMID: 37761831 PMC10531002

[B71] Waldman I. (1974). Letter: pneumothorax from acupuncture. N Engl. J. Med. 290, 633. doi: 10.1056/NEJM197403142901125, PMID: 4812510

[B72] Weeres M. A. Robien K. Ahn Y. O. Neulen M. L. Bergerson R. Miller J. S. . (2014). The effects of 1,25 dihydroxyvitamin D3 (1,25(OH)2D3) on *in vitro* human natural killer cell developmentFrom hematopoietic stem cells. J. Immunol. 193, 3456–3462. doi: 10.4049/jimmunol.1400698, PMID: 25149465 PMC4363084

[B73] Wejse C. Gomes V. F. Rabna P. Gustafson P. Aaby P. Lisse I. M. . (2009). Vitamin D as supplementary treatment for tuberculosis: a double-blind, randomized, placebo-controlled trial. Am. J. Respir. Crit. Care Med. 179, 843–850. doi: 10.1164/rccm.200804-567OC, PMID: 19179490

[B74] Wilkinson R. J. Llewelyn M. Toossi Z. Patel P. Pasvol G. Lalvani A. . (2000). Influence of vitamin D deficiency and vitamin D receptor polymorphisms on tuberculosis among Gujarati Asians in west London: a case-control study. Lancet 355, 618–621. doi: 10.1016/S0140-6736(99)02301-6, PMID: 10696983

[B75] Wingfield T. Schumacher S. G. Sandhu G. Tovar M. A. Zevallos K. Baldwin M. R. . (2014). The seasonality of tuberculosis, sunlight, vitamin D, and household crowding. J. Infect. Dis. 210, 774–783. doi: 10.1093/infdis/jiu121, PMID: 24596279 PMC4130318

[B76] Wu Q. Liu Y. Ma Y. B. Liu K. Chen S. H. (2022). Incidence and prevalence of pulmonary tuberculosis among patients with type 2 diabetes mellitus: a systematic review and meta-analysis. Ann. Med. 54, 1657–1666. doi: 10.1080/07853890.2022.2085318, PMID: 35703920 PMC9225779

[B77] Yadav U. Kumar P. Rai V. (2021). FokI polymorphism of the vitamin D receptor (VDR) gene and susceptibility to tuberculosis: Evidence through a meta-analysis. Infect. Genet. Evol. 92, 104871. doi: 10.1016/j.meegid.2021.104871, PMID: 33901685

[B78] Yao M. Oduro P. K. Akintibu A. M. Yan H. (2024). Modulation of the vitamin D receptor by traditional Chinese medicines and bioactive compounds: potential therapeutic applications in VDR-dependent diseases. Front. Pharmacol. 15. doi: 10.3389/fphar.2024.1298181/full, PMID: 38318147 PMC10839104

[B79] Zhang J. Chen C. Yang J. (2019). Effectiveness of vitamin D supplementation on the outcome of pulmonary tuberculosis treatment in adults: a meta-analysis of randomized controlled trials. Chin. Med. J. (Engl) 132, 2950–2959. doi: 10.1097/CM9.0000000000000554, PMID: 31833904 PMC6964947

[B80] Zhang J. W. Zhang Q. Qu D. B. Lin Z. Ma X. M. Zhong X. . (2017). Association of vitamin D receptor gene polymorphisms with susceptibility to bone and joint tuberculosis in Chinese Han population. Nan Fang Yi Ke Da Xue Xue Bao. 37, 704–706. doi: 10.3969/j.issn.1673-4254.2017.05.24, PMID: 28539299 PMC6780463

